# Systematic screening of 42 vancomycin-resistant *Enterococcus faecium* strains for resistance, biofilm, and desiccation in simulated microgravity

**DOI:** 10.1038/s41526-024-00447-8

**Published:** 2024-11-13

**Authors:** Franca Arndt, Katharina Siems, Sarah V. Walker, Noelle C. Bryan, Stefan Leuko, Ralf Moeller, Alessa L. Boschert

**Affiliations:** 1 Institute of Aerospace Medicine, Aerospace Microbiology, German Aerospace Center (DLR), Cologne, Germany; 2 Institute for Medical Microbiology, Immunology and Hygiene, University Hospital of Cologne, Cologne, Germany; 3 Department of Cardiac Surgery, Brigham and Women’s Hospital, Boston, MA USA

**Keywords:** Microbiology, Infectious diseases

## Abstract

Vancomycin-resistant *Enterococcus faecium* (VRE) presents significant challenges in healthcare, particularly for hospitalized and immunocompromised patients, including astronauts with dysregulated immune function. We investigated 42 clinical *E. faecium* isolates in simulated microgravity (sim. µ*g*) using a 2-D Clinostat, with standard gravity conditions (1 *g*) as a control. Isolates were tested against 22 antibiotics and characterized for biofilm formation and desiccation tolerance. Results showed varied responses in minimum inhibitory concentration (MIC) values for seven antibiotics after sim. µ*g* exposure. Additionally, 55% of isolates showed a trend of increased biofilm production, and 59% improved desiccation tolerance. This investigation provides initial insights into *E. faecium’s* changes in response to simulated spaceflight, revealing shifts in antibiotic resistance, biofilm formation, and desiccation tolerance. The observed adaptability emphasizes the need to further understand VRE’s resilience to microgravity, which is crucial for preventing infections and ensuring crew health on future long-duration space missions.

## Introduction

The antimicrobial resistance crisis is a worldwide health threat, which poses the risk of losing the effectiveness of antibiotics and, therefore, lifesaving therapy options in the near future. According to the Centers for Disease Control and Prevention around 35,900 deaths, and over 2 million infections, can be ascribed to antimicrobial-resistant bacteria and fungi in the USA alone. The so called “ESKAPE pathogens” play a key role in the global challenge of over- and misuse of antibiotics^1^. This group of bacteria, including *Enterococcus (E.) faecium, Staphylococcus aureus* (*S. aureus*), *Klebsiella pneumoniae, Acinetobacter baumannii, Pseudomonas aeruginosa* (*P. aeruginosa*), and Enterobacter species, can be multi-drug resistant, enabling them to survive antibiotic treatments^2^. They are the major cause of healthcare-associated infections resulting in ~33,000 deaths and 874,000 disability-adjusted life-years in Europe alone^3,4^.

*E. faecium* is a biosafety level 2 bacterium and an opportunistic pathogen of the gastrointestinal flora, intrinsically resistant to numerous antibiotics. In addition, *E. faecium* may become resistant to vancomycin, a first-line antibiotic for many infections. This resistance for glycopeptide antibiotics is mediated by van genes, with *vanA* and *vanB* being highly relevant, as they can both be plasmid-encoded. The vancomycin-variable Enterococci (VVE) harbor either the *vanA* or *vanB* gene complex, exhibiting a vancomycin-resistant genotype with a susceptible phenotype^5,6^. If VVE strains are exposed to vancomycin during infection and subsequent therapy, induction of vancomycin resistance may occur^7^. VVE strains carrying the *vanB* (VVE-B) gene were included in this study.

In the hospital setting and particularly in immunocompromised patients^8^, VRE causes various severe infectious complications, such as bacteremia and endocarditis^9,10^. Especially biofilm associated infections, such artificial valve endocarditis, often further complicate treatment^11–13^. With an additional resistance to vancomycin, reserve antibiotics, such as linezolid and daptomycin, are the only therapy option left for VRE. In addition, their great tenacity, including high desiccation tolerance^14^, results in VRE being a particularly relevant pathogen for (endemic) hospital outbreaks^15^.

VRE pose a significant threat, not only to immunocompromised patients but also to astronauts, given their dysregulated immune function during spaceflight, which makes them more susceptible to infections^16,17^. Astronauts have to endure various physiological challenges on board the International Space Station (ISS). These include, but are not limited to muscle atrophy and loss of bone density^18^, the increased risk of cancer^19^, psychological stress^20^, altered sleep patterns^21^, and the spaceflight-associated neuro-ocular syndrome^22^.

During spaceflight, the gastrointestinal microbiome of astronauts are exposed to the harsh environment, exposing them to stress such as microgravity and increased radiation^23,24^. The actual presence of antimicrobial resistances already poses a serious infections risk in a built environment like the ISS, even in close proximity to Earth^25^. For example, during the Apollo 13 mission, a crew member experienced an in-flight ESKAPE pathogen infection of *P. aeruginosa*^26^. This underscores the significant challenges posed by infections in the confined and isolated environments of spacecrafts with only limited medical resources. This scenario may be further aggravated by infections caused by multi-drug resistant bacteria. Due to the built environment and the dysregulated immune functions in spaceflight some similarities can be drawn between the hospital setting and the spaceflight environment, including a potential risk for VRE infection.

The query of limited treatment of infections is even further intensified by the already planned long-term missions to the Moon, such as the NASA Artemis missions, and – potentially – to Mars^27^. This is of particular importance for potential pathogenic members of the gut microbiomes, including VRE – that are part of the physiological human microbial flora and can cause infections under specific circumstances, including dysregulated immune function^28,29^. The unavoidable carrying along of the human microbiota, including *E. faecium*, in combination with a confined environment, limited treatment options and multiple external stress factors highlight the crucial need for understanding antibiotic resistance of VRE under spaceflight conditions. Particularly in the field of human spaceflight, the research is still evolving and knowledge gaps remain regarding drug stability and how antibiotic resistances may change in space during long-term missions^30^. However, changes in bacterial resistance still require further investigation, since different studies show increases, decreases or no changes in minimal inhibitory concentrations (MIC) due to simulated microgravity. Overall, there seems to be no general “spaceflight response” of bacteria and the adaptations vary depending on the bacterial species^31–33^. This fact highlights the need to gain further insights into bacterial changes to microgravity and built environment in order to ensure future safeguarding of astronauts’ health and gaining valuable insights to address the global antibiotic resistance crisis^34^.

Since spaceflight experiments are highly demanding in terms of planning time, costs, and engineering, and allow only a limited number of experiments to be conducted, ground-based models and experiments are essential. These models provide low-shear environments that offer a preliminary approximation of potential changes during actual spaceflight. However, the direct transferability of results to microgravity conditions may be limited and should be approached with caution. Furthermore, comparisons between ground-based experiments are hindered by the variety of simulation systems available. Different systems, such as rotation vessel culture apparatus, can be used: rotary cell culture system, high-aspect ratio vessel, or rotating-wall bioreactor, which are often employed for cultures and incorporate a hydrophobic membrane for aeration. Additionally, slow-turning lateral vessel and integrated rotating-wall vessel are also applicable^35^. These systems provide low fluid shear forces, replicating the loss of gravity through the rotation of cells, which nullifies gravity vectors. Other options for simulating microgravity include random positioning machines and clinostats.

2D-clinostats have been used in several studies with bacteria^36,37^. They are easy to operate within a simple setup and are accessible to a wide range of experiments. The uniform gravity cancellation across the entire sample ensures consistent experimental conditions. This is of especial importance for experiments necessitating prolonged exposure periods^38^.

In contrast to slow-rotating clinostats which focus on overall geometry to meet a predetermined condition, fast-rotating clinostats also account for the sedimentation paths in a fluid. Hence, higher speeds (60 r.p.m) may be used for high viscosity media, like agar and adherent cells^39–41^. With bacterial colonies growing in the center of the agar plates in alignment with the horizontal axis and not exceeding a radius of 1 cm, arising centrifugal and acceleration forces can be neglected: a colony radius of 0.3–0.4 cm, *g*-forces of 1.2 × 10^−2^–1.6 × 10^−2^ *g*, ensure exposure to simulated microgravity of the whole colony based on the following calculation of simulated µ*g*: *a* = ω 2 *r* [*ω*: angular velocity, *r*: colony radius]^39^. The Coriolis force *gf* = 2*ωϕ* [*g*: gravity, *ω*: angular velocity, *ϕ*: angle per unit time] is proportional to the velocity of the microorganism and the rotation speed^40^. Previous studies have shown, that at 60 r.p.m, the resulting Coriolis force is extremely small and insignificant for microorganisms due to their low velocity and mass^40,42^.

Static 1 *g* controls placed directly next to the clinostat in order to ensure similar humidity and temperature conditions, have been established as a standard practice in clinostat experiments^36,37^. This ensures, that the observed effects are due to microgravity rather than other factors.

When studying bacterial responses to sim. µ*g*, the medium in which they are cultured plays a crucial role. Bacterial cells respond differently to sim. µ*g* on solid media compared to liquid media due to factors like cell-to-surface interactions, nutrient diffusion, shear stress, and altered gene expression. For example*, Salmonella enterica* shows changes in stress response and metabolism gene expression^43^. Solid surfaces allow bacteria to sense microgravity through different environmental interactions, affecting horizontal gene transfer^44^. Microgravity can alter biofilm architecture and gene expression, highlighting the importance of studying both solid and liquid media to understand these effects^42^. Solid media provide a stable, low-shear environment, crucial for reliable biofilm studies and consistent experimental results. Therefore, we chose this setup to ensure the reliability and consistency of our experimental results.

The impact of microgravity on microbial fitness, tolerances to harsh environments and antibiotic efficacy still has to be determined for Enterococci. Our study aimed to screen for changes in antibiotic susceptibility, biofilm formation and desiccation tolerance for 42 *E. faecium* isolates under sim. µ*g*. Due to our need to use solid media and an exposure to microgravity over 7 consecutive days, we decided for the clinostat as the best option for our setup. This research is essential for ensuring the health of astronauts during long-duration space missions, which last from several months to over a year, and for developing effective strategies to combat antibiotic resistance. Additionally, it may not only benefit human spaceflight but may also result in new strategies in healthcare settings.

## Results

### Antibiotic susceptibility after sim. µ*g*

All *E. faecium* isolates (VRE *n* = 15, VVE-B *n* = 7, VSE *n* = 20, total = 42) were evaluated for their antibiotic susceptibility, before (initial) and after incubation under sim. µ*g* or 1 *g* conditions. With the exclusion of strains VRE-6, -8, -10, and -13, all isolates were tested in triplicate for their susceptibility to 22 antibiotics (Supplementary Material, Table 3, Fig. 8). The third replicate (Rep. 3) was excluded for the above isolates due to contamination (Supplementary Information). Isolates that showed a single-step increase or decrease in MIC after exposure to sim. µ*g* (or after 1 *g*) are listed in the Supplementary Information (Supplementary Table 1). These one step changes in MIC were observed for 59% (13/22) of the tested antibiotics (ampicillin, daptomycin, fosfomycin, fusidic acid, gentamycin, high level gentamycin, linezolid, mupirocin, rifampicin, quinupristin/dalfopristin, trimethoprim/sulfamethoxazol, teicoplanin, vancomycin). Changes to those antibiotics occurred in 85% (36/42) of the isolates (VRE *n* = 11, VSE *n* = 19, VVE-B *n* = 6; Supplementary Table 1). For cefoxitin, tigecylin, erythromycin/clindamycin and clindamycin, no changes were observed for any tested isolates at all in MIC.

Identical MIC after incubation in sim. µ*g* and 1 *g* were observed in 30% of the strains (13/42; VRE *n* = 6, VSE *n* = 4, VVE-B *n* = 3) (Supplementary Table 2) for nine different antibiotics (ampicillin, daptomycin, erythromycin, fosfomycin, penicillin, rifampicin, quinupristin/dalfopristin, trimethoprim/sulfamethoxazole, vancomycin). Out of these strains, the highest increase in MIC was observed in two VVE-B and one VRE isolate: the strains VVE-B-16 and -19 increased four steps in their MIC for erythromycin and vancomycin after incubation under sim. µ*g* and 1 *g*, respectively. Isolate VRE-8 increased its teicoplanin MIC from 0.25 to >16 µg/mL (five steps) after incubation under sim. µ*g* and 1 *g* (Supplementary Table 2). The highest reduction in MIC in both conditions (sim. µ*g*, 1 *g*) was observed for isolate VSE-35, which decreased from 8 to 0.5 µg/mL to vancomycin. This isolate displayed MIC changes in a total of six antibiotics (ampicillin, ceftarolin, moxifloxacin, penicillin G, rifampicin, vancomycin) after incubation in sim. µ*g* and 1 *g*. This was the highest number of affected antibiotics out of all isolates. All MIC changes in strain VSE-35 ranged between one and three steps in MIC reductions.

Isolates with altered antibiotic susceptibility after incubation under sim. µ*g*, which were not identical with the 1 *g* control are shown in Table 1. Each of the seven isolates (VRE *n* = 2, VSE *n* = 3, VVE-B *n* = 2) changed in their MIC to different antibiotics (ceftarolin, gentamycin, moxifloxacin, oxacillin, rifampicin, quinupristin/dalfopristin, vancomycin) (Table 1). Out of these seven isolates, four decreased and three isolates increased in MIC after sim. µ*g*. The MIC of the isolate VRE-2 to gentamicin increased after sim. µ*g* (initial: 1 µg/mL, sim. µ*g*: 4 µg/mL), whereas the initial MIC was concurring with the MIC of the control (1 *g*: 1 µg/mL). VRE-4 decreased one step in its MIC after exposure to sim. µ*g* against quinupristin/dalfopristin, however increased in MIC in the control (sim. µ*g*: <0.5 µg/mL, 1 *g*: 4 µg/mL). The MIC for oxacillin reduced three steps after sim. µ*g* for strain ATCC 6057 while remaining constant under 1 *g* conditions (Table 1). The MIC to ceftarolin of isolate VSE-35 was reduced from >2 to 1 µg/mL after sim. µg and from >2 to 0.5 µg/mL after 1 *g*. In addition, the MIC of VSE-35 to moxifloxacin decreased after exposure to sim. µ*g* (initial: >2 µg/mL, sim. µ*g*: <0.25 µg/mL), as in 1 *g* (1 *g*: 0.5 µg/mL). Furthermore, isolate VVE-B-17 increased due to sim. µ*g* from 8 to 32 µg/mL but decreased to 1 µg/mL under 1 *g* conditions to vancomycin (Table 1). Isolate VVE-B-22 increased in the MIC to rifampicin after sim. µ*g* (initial: 0.5 µg/mL, sim. µ*g*: >2 µg/mL), remaining unaltered in the 1 *g* control (1 *g*: 0.5 µg/mL) (Table 1).

**Table 1:** Changes of antibiotic susceptibility after sim. µ*g* and 1 *g* in *E. faecium* isolates

Changes in MIC (µg/mL)				
Isolate	Initial	Sim. µ*g*	1 *g*	Antibiotic
VRE-2	1	4	1	Gentamycin
VRE-4	1	<0.5	>4	Quinupristin/dalfopristin
ATCC 6057	>16	>2	>16	Oxacillin
VSE-35	>2	1	0.5	Ceftarolin
VSE-35	>2	<0.25	0.5	Moxifloxacin
VVE-B-17	8	32	1	Vancomycin
VVE-B-22	0.5	>2	0.5	Rifampicin

Alterations are displayed in minimal inhibitory concentration MIC (µg/mL) for each tested antibiotic. All isolates were tested in triplicates.

*Initial* initial MIC of isolate, *sim. µg* MIC after incubation under sim. µ*g* for 7 days, *1* *g (control)* MIC after incubation for 7 days, Isolate: *VRE* vancomycin-resistant *E. faecium*, *VVE-B* vancomycin-variable *E. faecium* VanB type, *VSE* vancomycin susceptible *E. faecium*.

### Adherence of VRE after sim. µ*g*

**Fig. 1:**
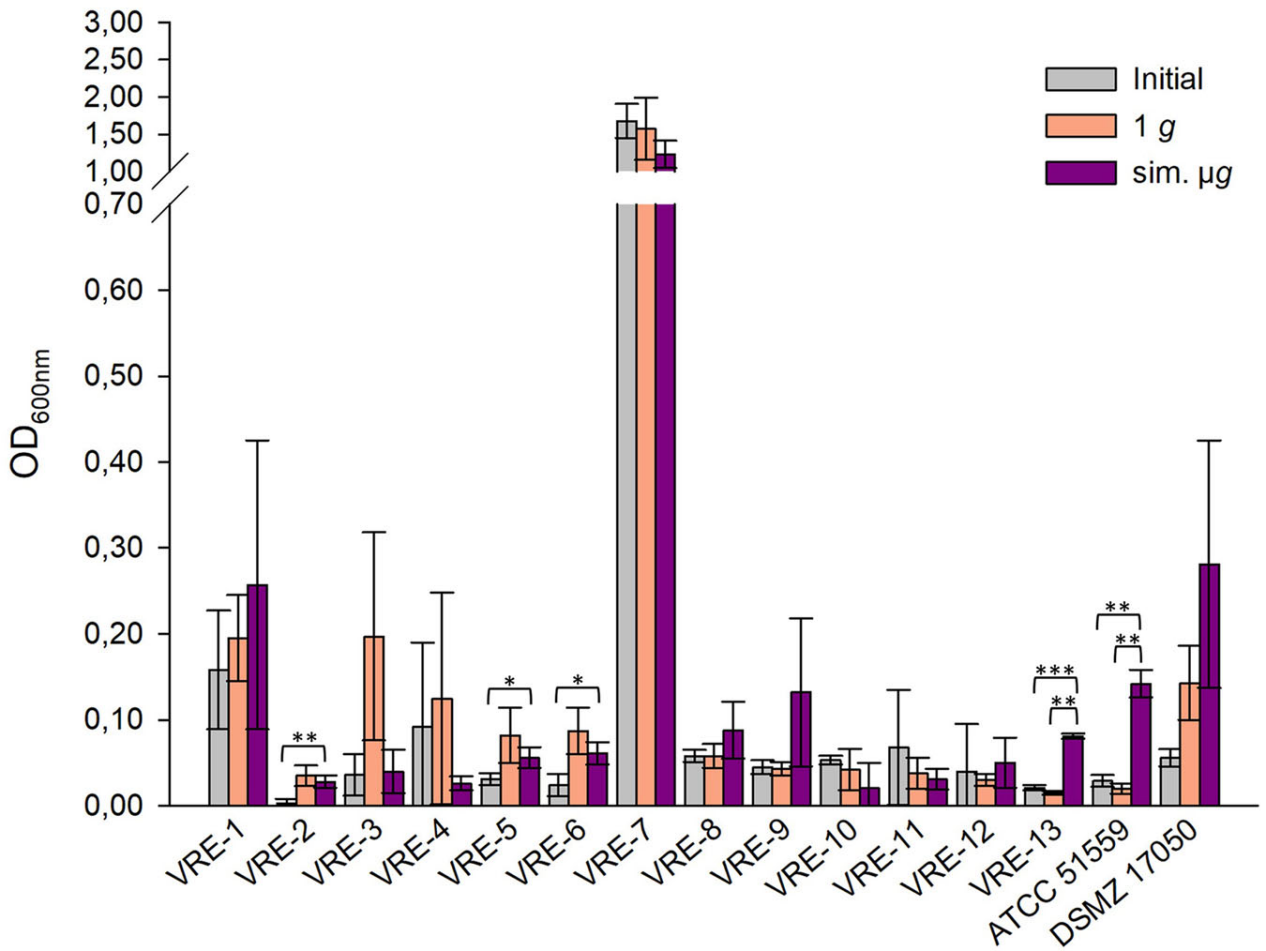
Adherence of VRE (vancomycin-resistant *E. faecium*) isolates tested with crystal violet (0.5%) biofilm assay (*n* = 15). Initial: adherent cell formation was initially evaluated with the crystal violet assay before any treatment. 1 *g*: isolates were incubated in normal gravity as a control for 7 days. Sim. µ*g*: isolates were incubated under sim. µ*g* for 7 days by clinorotation. Isolates were then tested in their ability to adhere and potentially form biofilms after 24 h at 37 °C in measuring the optical density (OD_600nm_) in a microplate reader. Measurements were done in triplicates and calculated error bars show the standard deviation of each sample (*n* = 3). Significance was determined by two-sample *t*-test; the conditions corresponding to the significance levels are shown in the parentheses: *p < 0.05; **p < 0.01; ***p < 0.001.

The adherent cells of VRE isolates ranged from low (OD_600nm_ = 0.03, VRE-2) to high (OD_600nm_ = 1.68, VRE-7) (Fig. 1). Among all VRE isolates, VRE-7 showed the highest adherence (initial: OD_600nm_ = 1.68, 1 *g*: OD_600nm_ = 1.57, sim. µ*g*: OD_600nm_ = 1.23). Five isolates exhibited significant less adherence to in the initial testing compared to the exposure to sim. µ*g* (VRE-2, -5, -6, -13, ATCC 15559) (Fig. 1). Additionally, isolates VRE-2, -3, and -6 showed significant decrease in the initial testing of adherent cells compared to the 1 *g* control (VRE-2 *p* = <0.05; VRE-3 *p* = <0.001; VRE-6 *p* = <0.05) (Fig. 1). Forty-six percent (7/15 isolates) displayed more adherent cells when exposed to sim. µ*g*. Of these, two isolates demonstrated a significant change in adherence following incubation under sim. µ*g* (VRE-13 *p* = <0.01; ATCC 51559 *p* = <0.01) when compared to the control (1 *g*) (Fig. 1).

**Fig. 2:**
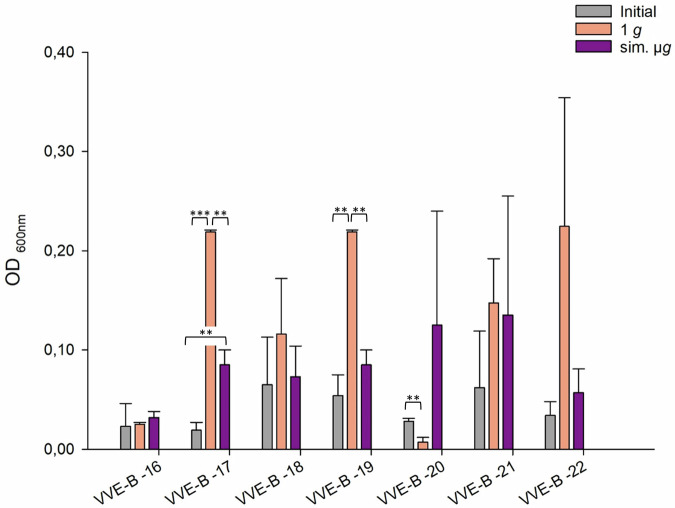
Adherence of VVE-B (vancomycin-variable *E. faecium*) isolates tested with crystal violet (0.5%) biofilm assay (*n* = 7). Initial: adherent cell formation was initially evaluated with the crystal violet assay before any treatment. 1 *g*: isolates were incubated in normal gravity as a control for 7 days. Sim. µ*g*: isolates were incubated under sim. µ*g* for 7 days by clinorotation. Isolates were then tested in their ability to adhere and potentially form biofilms after 24 h at 37 °C in measuring the optical density (OD_600nm_) in a microplate reader. Measurements were done in triplicates and calculated error bars show the standard deviation of each sample (*n* = 3). Significance was determined by two-sample *t*-test; the conditions corresponding to the significance levels are shown in the parentheses: **p < 0.01; ***p < 0.001.

Among the five VVE-B isolates tested, isolate VVE-B-17 showed significant less adherent cell formation in the initial testing compared to the adherent cells detected after sim. µ*g*. Isolates VVE-B-17 and -19 showed significant more adherence in the 1 *g* control than in the initial testing. Only isolate VVE-B-20 exhibited singinficant less adherence in the 1 *g* control compared to the initial testing (Fig. 2). Two isolates (VVE-B-16, VVE-B-20) exhibited increased adherent cells following exposure to sim. µ*g* (Fig. 2). Three isolates displayed a higher adherence in the control (1 *g*). Among these, two isolates (VVE-B-17, VVE-B-19) showed a significant decrease in adherent cells after incubation under sim. µ*g* (Fig. 2, VVE-B-17 *p* = <0.01; VVE-B-19 *p* = <0.01) than after 1 *g*. The highest number of adhered cells was observed in isolate VVE-B-17 and VVE-B-19 after incubation without sim. µ*g* (1 *g*, OD_600nm_ = 0.22) (Fig. 2).

**Fig. 3:**
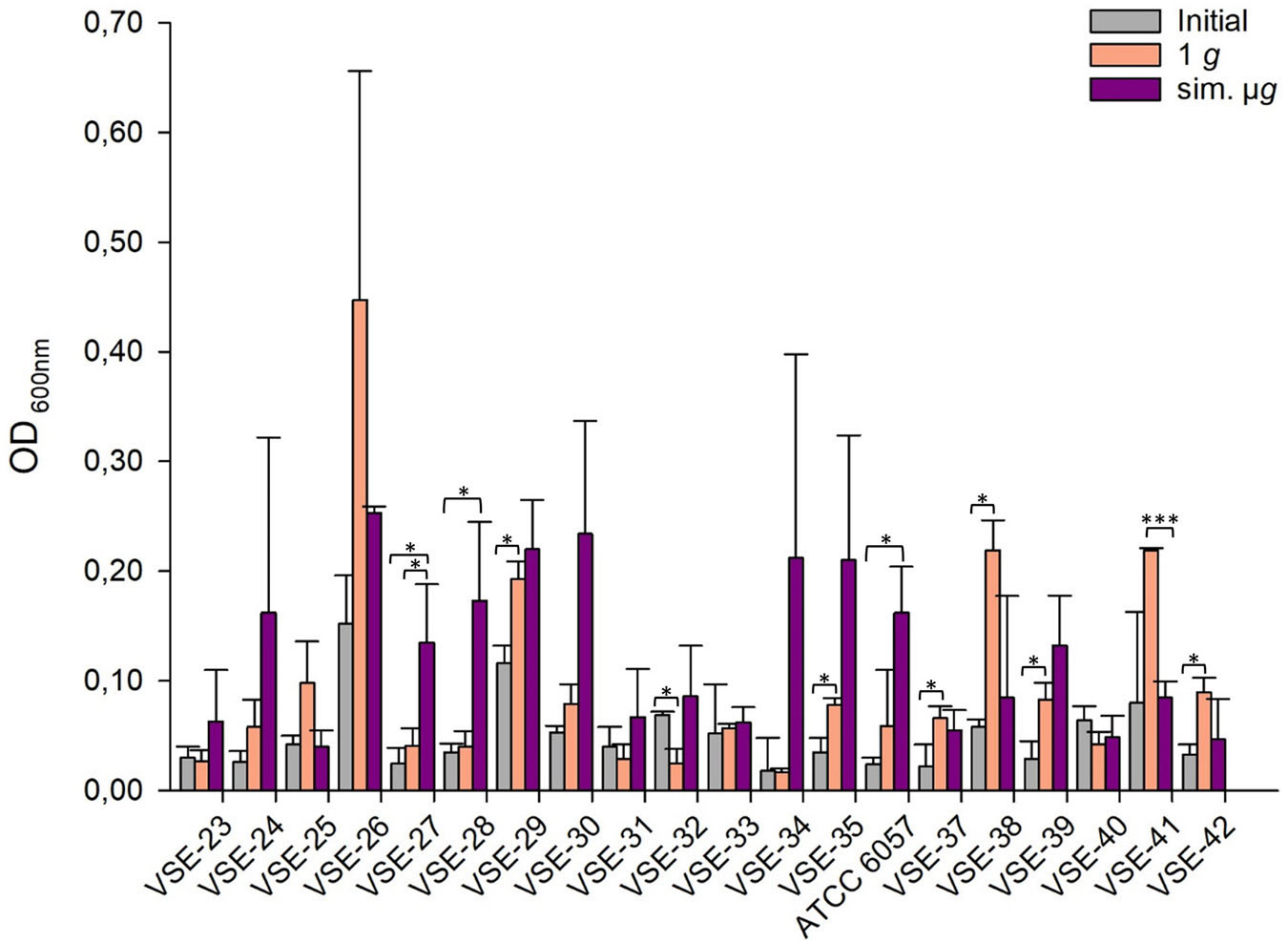
Adherence of VSE (vancomycin susceptible *E. faecium*) isolates tested with crystal violet (0.5%) biofilm assay (*n* = 20). Initial: adherent cell formation was initially evaluated with the crystal violet assay before any treatment. 1 *g*: isolates were incubated in normal gravity as a control for 7 days. Sim. µ*g*: isolates were incubated under sim. µ*g* for 7 days by clinorotation. Isolates were then tested in their ability to adhere and potentially form biofilms after 24 h at 37 °C in measuring the optical density (OD_600nm_) in a microplate reader. Measurements were done in triplicates and calculated error bars show the standard deviation of each sample (*n* = 3). Significance was determined by two-sample *t*-test. The conditions corresponding to the significance levels are shown in the parentheses: *p < 0.05; ***p < 0.001.

The VSE isolates exhibited differences in the number of adherent cells, with values ranging from OD_600nm_ = 0.17 (1 *g*, VSE-34) to OD_600nm_ = 0.447 (1 *g*, VSE-26). VSE-27 and ATCC 6057 had a significant increased adherence after sim. µ*g* compared to the initial testing (Fig. 3). Among all tested isolates 65% (13/20 isolates) showed an increased number of adherent cells after exposure to sim. µ*g* compared to 1 *g* (Fig. 3). Out of these, two isolates (VSE-27, VSE-28) exhibited significantly higher adherence compared to the control (VSE-27 *p* = <0.05; VSE-28 *p* = <0.05). Conversely, less adherence was observed for VSE-41 after exposure to sim. µ*g* compared to the control. Overall, 30% (6/20 isolates) displayed higher number of adherent cells after 1 *g* (without sim. µ*g*) (Fig. 3).

Of all tested isolates, 55% (23/42 isolates) had more adherent cells after sim. µ*g* in comparison to the 1 *g* control. Among these, four isolates demonstrated significantly higher adherence than in the 1 *g* control (VSE-27 *p* = 0.042; VSE-28 *p* = 0.035, VRE-13 *p* = <0.001; ATCC 51559 *p* = <0.001). Moreover, for 45% (19/42 isolates) of the isolates, adherence was even higher in the control (1 *g*). Among these, three isolates showed a significant increase in adherence (VSE-41 *p* = <0.001, VVE-B-17 *p* = <0.001; VVE-B-19 *p* = <0.001). In total, the abundance of adherent cells after sim. µ*g* was higher in VSE compared to VRE and VVE-B isolates. Sixty-five percent of the VSE isolates showed an increased adherence after sim. µ*g*, whereas it was higher under 1 *g* conditions for 59% of the VRE and 60% of the VVE-B isolates. Overall, five VRE isolates, one VVE-B isolate and two VSE isolates showed less adherence in the initial testing compared to the exposure to sim. µ*g*.

### Desiccation tolerance of VRE after sim. µ*g*

**Fig. 4:**
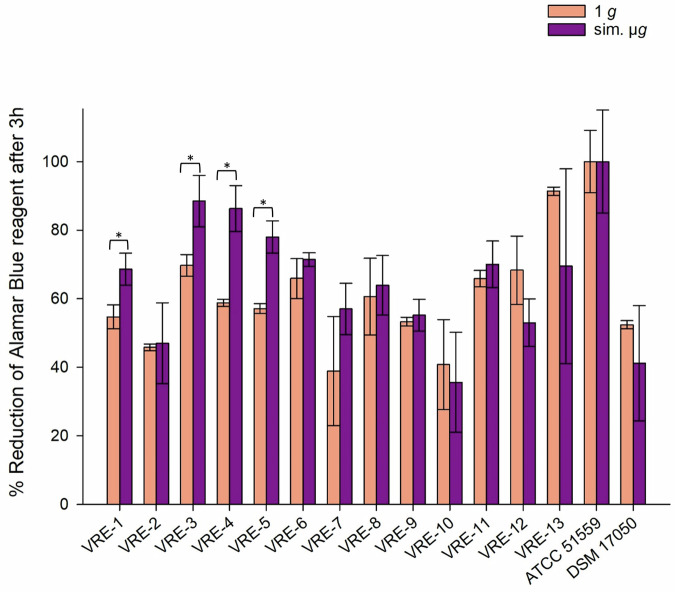
Reduction (%) of alamarBlue Cell Viability Reagent after 3 h incubation with VRE (vancomycin-resistant *E. faecium*) isolates after 24 h of desiccation. Isolates were incubated in normal gravity (1 *g*) and under sim. µ*g* for 7 days (*n* = 15). These isolates were then dried for 24 h and tested in their viability after desiccation by the Cell Viability Reagent alamarBlue. Measurements were done in triplicates and calculated error bars show the standard deviation of each sample (*n* = 3). Due to technical constraints, the assay was designed without the initial measurement to ensure that all samples fit within a single run of the measurement, maintaining consistency and accuracy across all data points. Significance was determined by two-sample *t*-test; The conditions corresponding to the significance levels are shown in the parentheses: *p < 0.05.

To measure the metabolic activity after sim. µ*g* and desiccation, the reduction of alamarBlue Cell Viability Reagent was measured. Higher values indicate greater cell viability. This correlates with increased survival after desiccation, thereby indicating higher desiccation tolerance. The reduction of alamarBlue Cell Viability Reagent for all VRE isolates (*n* = 15) after desiccation, both with and without sim. µ*g*, ranged from 35% (1 *g*, VRE-10) to 100% (sim. µ*g*, ATCC 51559). Seventy-three percent (11/15) of VRE isolates exhibited higher metabolic activity when exposed to sim. µ*g* compared to the control (1 *g*). Out of these, four VRE isolates displayed significantly increased viability after being incubated in sim. µ*g* (VRE-1 *p* = 0.015; VRE-3 *p* = 0.016; VRE-4 *p* = 0.002; VVE-B-21 *p* = 0.002). Five isolates (VRE-10, VRE-12, VRE-13, ATCC 51559, DSMZ 17050) showed higher metabolic activity after desiccation under 1 *g* conditions (Fig. 4).

**Fig. 5:**
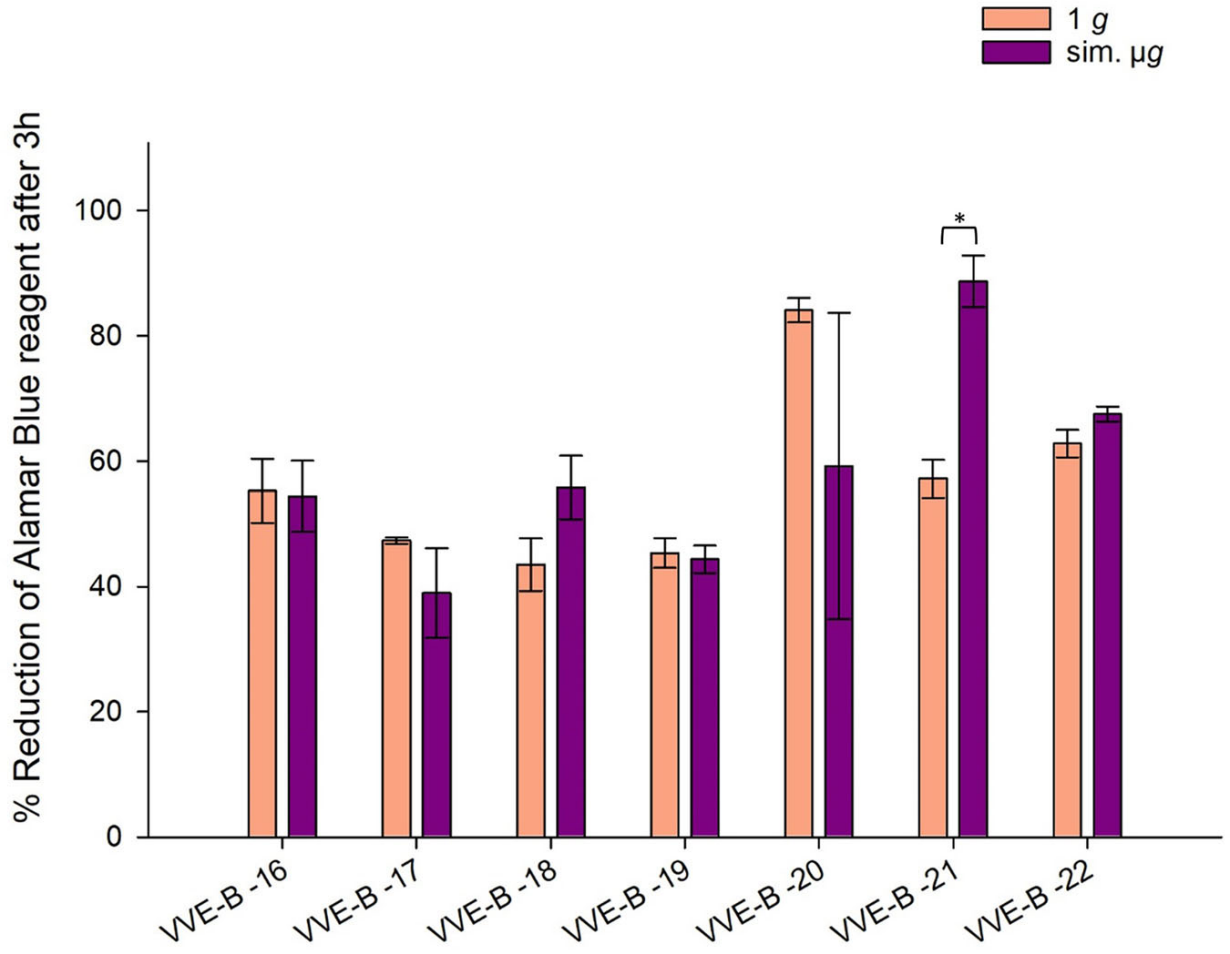
Reduction (%) of alamarBlue Cell Viability Reagent after 3 h incubation with VVE-B (vancomycin-variable *E. faecium*) isolates after 24 h of desiccation. Isolates were incubated in normal gravity (1 *g*) and under sim. µ*g* for 7 days (*n* = 15). These isolates were then dried for 24 h and tested in their viability after desiccation by the Cell Viability Reagent alamarBlue. Measurements were done in triplicates and calculated error bars show the standard deviation of each sample (*n* = 3). Due to technical constraints, the assay was designed without the initial measurement to ensure that all samples fit within a single run of the measurement, maintaining consistency and accuracy across all data points. Significance was determined by two-sample *t*-test. Significance was determined by two-sample *t*-test; The conditions corresponding to the significance levels are shown in the parentheses: *p < 0.05.

Six out of seven tested VVE-B isolates (VVE-B-16, -17, -18, -19, -20, -22), showed no significant changes in their desiccation tolerance, regardless of the incubation conditions. However, isolate VVE-B-21 exhibits significant higher metabolic activity when exposured to sim. µ*g* prior to desiccation (VVE-B-21 *p* = 0.0004) (Fig. 5).

The majority of the 20 tested VSE isolates demonstrated increased tolerance to desiccation after exposure to sim. µ*g*: among these, 70% (14/20 isolates) exhibited enhanced desiccation tolerance following sim. µ*g* exposure compared to the control. Four isolates showed statistically significant increased metabolic activity when incubated under sim. µ*g* (VSE-24 *p* = 0.026; VSE-25 *p* = 0.021; VSE-32 *p* = <0.001, VSE-39 *p* = 0.003). In contrast, two isolates displayed higher metabolic activity in the controls without incubation under sim. µ*g* (VSE-33 *p* = 0.032; VSE-34 *p* = 0.029).

**Fig. 6:**
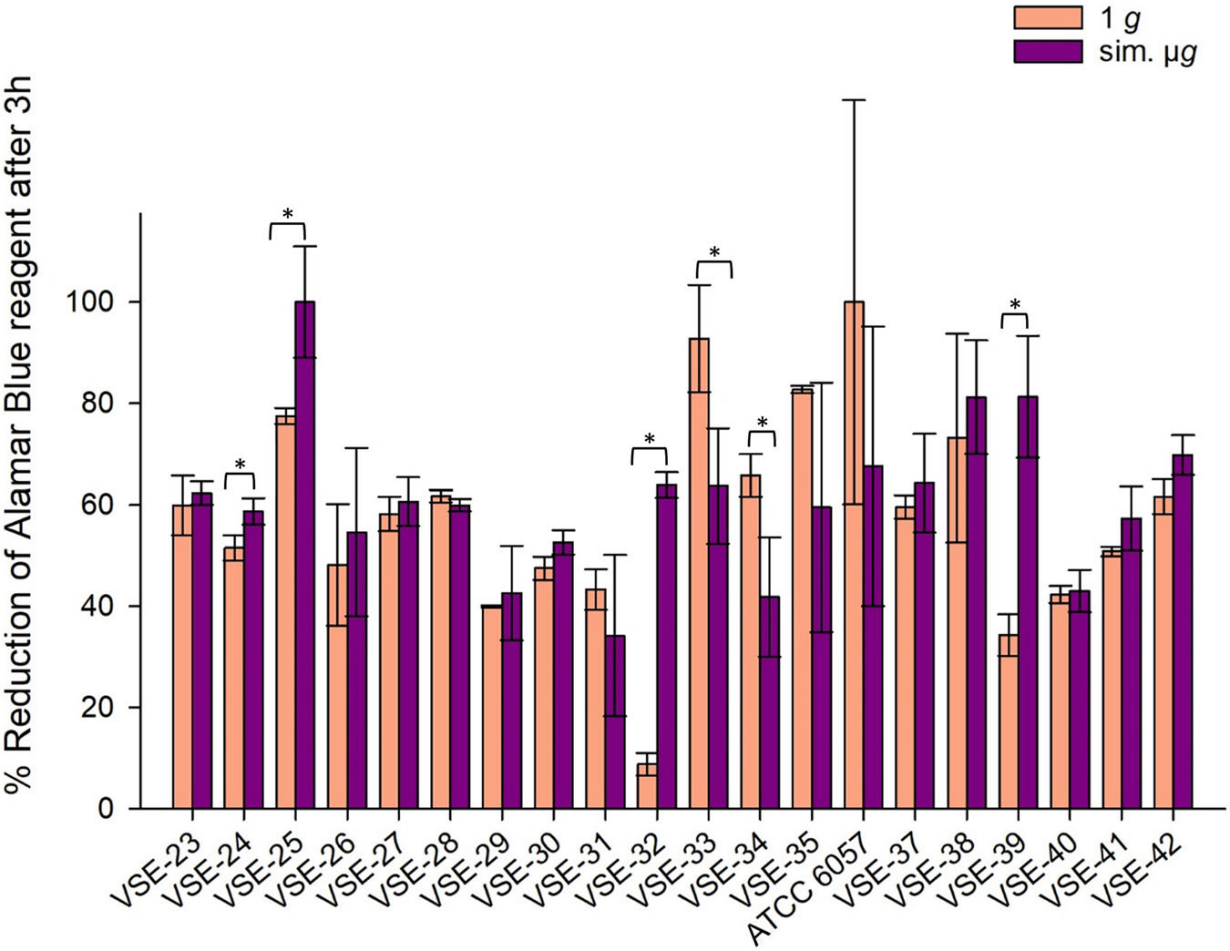
Reduction (%) of alamarBlue Cell Viability Reagent after 3 h incubation with VSE (vancomycin susceptible *E. faecium*) isolates after 24 h of desiccation. Isolates were incubated in normal gravity (1 *g*) and under sim. µ*g* for 7 days (*n* = 20). These isolates were then dried for 24 h and tested in their viability after desiccation by the Cell Viability Reagent alamarBlue. Measurements were done in triplicates and calculated error bars show the standard deviation of each sample (*n* = 3). Due to technical constraints, the assay was designed without the initial measurement to ensure that all samples fit within a single run of the measurement, maintaining consistency and accuracy across all data points. Significance was determined by two-sample *t*-test. Significance was determined by two-sample *t*-test; the conditions corresponding to the significance levels are shown in the parentheses: *p < 0.05.

The overall range of metabolic activity among all VSE isolates, after incubation with and without sim. µ*g* and following desiccation was 8.8% (1 *g*, VSE-32) to 100% (sim. µ*g*, VSE-25; 1 *g*, ATCC 6057) (Fig. 6). In summary both, VSE (70%) and VRE (64%) showed higher tolerance to desiccation after sim. µ*g* exposure than under normal gravity. Contrary to the VVE-B isolates, which showed no in- or decreased metabolic activity due to sim. µ*g*.

## Discussion

Overall, our findings indicate that *E. faecium* isolate are highly variable in their antibiotic susceptibility, desiccation tolerance, and biofilm formation under sim. µ*g*. As a result, there is no generalized adaption to sim. µ*g* that is transferrable to species level.

Several studies have investigated changes in antibiotic resistances under space conditions (e.g. *Escherichia* (*E*.) *coli, S. aureus*)^31,45^ and indicate an altered genotypic or phenotypic antibiotic resistance^46,47^. Here, a systematic investigation of therapeutically relevant antibiotics under spaceflight analog conditions has been conducted. This allows for novel insights into altered resistance selection and how this might differ between antibiotic groups and/or on a strain level.

All isolates (VRE, VSE, and VVE-B) showed changes in their MIC after cultivation in sim. µ*g*. This suggests that sim. µ*g* may select for strains carrying these traits, highlighting the potential for altered resistance under these conditions. While the underlying mechanism requires follow-up studies, our findings are in accordance with previous studies in which one original strain resulted in numerous populations with varying phenotypes^33^.

Of special interest is the observed vancomycin MIC increase in VVE-B isolate (VVE-B-17) under sim. µ*g* conditions due to two reasons: first, since vancomycin is the first-line therapy in most of invasive *E. faecium* infections, any MIC increases for this antibiotic should be considered highly critical. Second, a change in gene expression seems to be the possible mechanism in this particular case: as VVE-B already carry a *vanB* gene, expression changes can be considered more likely than a complete change in the genome itself. The increase in MIC suggests that the bacteria are becoming less susceptible to vancomycin, potentially leading to treatment failures. This is particularly alarming in a spaceflight environment where the immune functions of astronauts are already dysregulated, and the closed environment could facilitate the spread of resistant isolates. To understand the underlying mechanisms, genomic analysis is vital for follow-up studies.

Interestingly, changes in antibiotic susceptibility were also noted under normal gravity conditions after 7 days of incubation at 37 °C, which mimics body temperature. One-third of the isolates demonstrated altered antibiotic susceptibility to nine different antibiotics, indicating that prolonged incubation can induce resistance changes in *E. faecium* even without sim. µ*g*.

Changes in resistance were observed across six different antibiotic classes: glycopeptides (vancomycin), aminoglycosides (gentamicin), rifamycins (rifampicin), fluoroquinolones (moxifloxacin), beta-lactams (ceftaroline, oxacillin), streptogramins (quinupristin/dalfopristin). Some of these classes, such as aminoglycosides and rifamycins have shown to have synergistic effects in combination therapies – although lately cephalosporins have been favored over aminoglycosides due to a better side-effects profile^48^. However, no pattern throughout the different antibiotic classes was noted. Importantly, we could also not find a common response to sim. µ*g* for each of the respective groups – VRE, VVE-B, VSE. This variability suggests that *E. faecium* can alter both intrinsic and acquired resistances under sim. µ*g*, and that there is no universal resistance response within the species. In general, changes in antibiotic resistance profiles could be due to altered shear forces, mass diffusion rates, and osmotic gradients^35^. Understanding these mechanistic effects is crucial to ensure the health for astronauts on spaceflight missions and planned long-term future missions.

Various studies have investigated antibiotic resistances under spaceflight- or analog conditions and antibiotic-resistant bacteria have been detected on board of the ISS^49,50^. However, no VRE have been detected thus far. Nevertheless, vancomycin resistance genes and genomic signatures of the closely related species *Enterococcus faecalis* (*E*. *faecalis*) were found from a dining table on the ISS^51^. Moreover, 4.3% *E. faecalis* strains detected from the air on board the ISS were resistant to five out of nine tested antibiotics (chloramphenicol, erythromycin, high-level streptomycin, kanamycin, tetracycline)^52^. In another study, an *E. faecalis* strain, recovered from a steel surface after 12 months on the ISS, showed resistances to nine different antibiotics and was therefore, out of 78 isolates, the isolate with the maximum resistance profiles^53^. Additionally, 29 antimicrobial resistance genes were found on surfaces inside the ISS^54^ and 123 antimicrobial resistance genes were detected from 24 collected samples (over a 1-year period)^25^.

For gram-negative bacteria like *E. coli*, an increase in resistance in colistin and kanamycin was observed^45^. Another gram-negative bacterium, *P. aeruginosa*, showed also significant increased MICs to colistin in a spaceflight experiment^55^. Yet, comparability to our study is impeded due to those organisms being gram-negative and, therefore, have a different cellular membrane and wall structure. This not only means, that resistance mechanisms vary considerably, but also that different antibiotic classes were tested in the first place due to intrinsic resistances caused by these dissimilarities.

While comparability might be slightly improved when looking at gram-positive organisms, results are not readily transferrable between species. This, again, is due to different intrinsic resistances and resistance mechanisms. This should not be overlooked, given that our results already indicate tremendous differences within a species, and a comparison should be made carefully. Yet, because of a similar cellular construction, certain derivations may be possible when looking at different antibiotic classes. For instance, oxacillin MIC increased slightly in *S. aureus*^45^. In contrast, we observed a decreased resistance to oxacillin in a VSE isolate. However, apart from the already stated difference – oxacillin being an isoxazolyl penicillin and therefore one of the main therapy options for *S. aureus* infections while playing no role in treatment of *E. faecium* – our results occurred after exposure to sim. µ*g* and not during a spaceflight experiment, and, therefore, are not directly comparable.

Under sim. µ*g*, in a HARV, *S. aureus* showed no significant differences in susceptibility to erythromycin, flucloxacillin, or vancomycin^31^. Conversely, vancomycin MIC increased in some of our strains after exposure to sim. µ*g* by clinorotation. Furthermore, we detected an increased in MIC for erythromycin and vancomycin after both conditions in three isolates (VVE-B-16, -19, -20), incubation under sim. µ*g* and 1 *g*. Resistances against flucloxacillin were not investigated in our study. Yet, this underlines the already stated fact about not only interspecies but also intraspecies variability: as flucloxacillin also belongs to the group of isoxazolyl penicillins, a similar tendency as in the study of Tixador et al. should have been expected for *S. aureus*^45^. Moreover, gram-positive bacterium *Lactobacillus (L.) acidophilus*, showed significantly increased antibiotic resistance after sim. µ*g*, to cefalexin, sulfur gentamicin, and sodium penicillin^56^. However, in this experimental setup a 2-D RWV was used with a low rotation speed of 30 r.p.m., not 60 r.p.m. Lactic bacteria, such as *Lactobacillus* can also exhibit vancomycin resistance which is caused by a change from the D-Ala-D-Ala C-terminus of peptidoglycans to a D-Ala-D-Lac terminus^57^ – similar to the acquired resistance against vancomycin in VRE^58^. However, hybridization experiments failed, pointing toward a different resistance mechanism, such as a natural ligase, in *Lactobacillus* spp.^56^. This illustrates that phenotypic changes, even in gram-positive bacteria, cannot be fully understood without further insights into their underlying mechanisms. In conclusion, the different results stress the importance of understanding antimicrobial resistances in highly relevant species under spaceflight or analog conditions. When comparing studies concerning antibiotic resistance, it is important to note that they vary from actual spaceflight experiments, and many different test systems are used when simulating microgravity. Additionally, the studies differ in model organisms (gram-positive or gram-negative) and the antibiotics tested, which makes a close comparison of our results difficult. Further, they stress the need for understanding the observed changes in each of the species and strains in a molecular level.

For immunosuppressed patients as well as astronauts on the ISS, biofilms present a great health hazard^59,60^. They can maintain a continuous infection focus that, again, can provoke hospital-acquired infections such as bacteraemia and urinary tract infections^61^. Ongoing research on biofilm formation under varying gravitational conditions is necessary to fully understand the impact of gravity – especially for clinically relevant species such as *E. faecium*. In our study, using adherent cell measurements as a surrogate parameter for biofilm formation, the highest biofilm levels were observed in VRE isolates under both sim. µ*g* and normal gravity, supporting the link between antibiotic-resistant *E. faecium* and increased biofilm production^62^. Biofilm production was generally higher under normal gravity than sim. µ*g* for both VRE and VVE-B isolates, suggesting prolonged incubation supports biofilm formation. Recent literature shows adaptations in stressed biofilms due to nutrient depletion^24,63^. No similar literature has been found that supports or aligns with our findings regarding increased biofilm production solely due to prolonged incubation. Against our expectations, we did not observe any common phenotypes or any isolates standing out regarding development of increased biofilm and antimicrobial resistance. Of interest, strain VRE-7 seemed to produce notably more biofilm under all tested conditions when compared to the other strains. No common phenotype was found within VVE-B, VRE, and VSE. Overall, our results clearly show that *E. faecium* is able to alter in its adherence and therefore potentially in biofilm formation after exposure to sim. µ*g* (regardless if it is in- or decreased biofilm).

Sobisch et al.^53^ assessed the biofilm production of several Enterococci isolates and classified the ISS isolate of a multi-drug resistant *E. faecalis* as a strong biofilm former^53^. Other space related experiments showed an increased biofilm production of *P. aeruginosa*^64^ and thicker biofilms with higher tolerance to stressors (salt, ethanol) after sim. µ*g* in *E. coli*. Moreover, as expected, those biofilms were found to be more resistant to certain antibiotics^65^. Especially in biofilms, the dissemination of plasmids carrying antibiotic resistance genes is very likely^66^. Yet, not least because of various Earth-based modeling systems used to study the effects of microgravity on microorganisms, there still remain many open questions about biofilm formation under spaceflight conditions^38^.

Enterococci are highly tolerant to desiccation, surviving several months on dry surfaces like glass, stainless steel, plastic, and cloth^67^. This contributes to their environmental persistence and increases the risk of transmission among hospital patients and astronauts on the ISS, since both are confined spaces with limited access, which amplifies this risk. So far, there was no difference found between VSE and VRE regarding the survival on dry surfaces^68^. This corresponds with our findings, since we found no significant differences in metabolic activities for VSE and VRE. Under desiccation, VSE and VRE may more effectively express stress response genes that enhance survival mechanisms like DNA repair, protein stabilization, and membrane integrity maintenance. These desiccation resistance genes might be upregulated in VSE and VRE but not in VVE-B. Since the genes responsible for desiccation tolerance in Enterococci are not well understood, a genomic analysis of nanopore sequenced isolates is currently underway to investigate genes involved in this process. A study found that the desiccation tolerance of *E. faecalis* isolates from ISS did not differ from control isolates^69^, suggesting that the extreme environment on the ISS is not necessarily selecting for desiccation tolerant isolates. This aligns only partially with our results, as we found individual isolates that increased in desiccation due to sim. µ*g*. Therefore, our results provide initial insights into desiccation tolerance in *E. faecium* and its potential relation to gravity loss. Here, we demonstrated that *E. faecium* can change in its desiccation tolerance on dry surfaces under sim. µ*g* conditions, yet the exact underlying mechanisms remain unclear.

One limitation that needs to be considered when looking at the results of our study is the fact that the final testing was carried out under 1 *g* conditions. However, this constraint, caused by currently available technology and assays, is not limited to our study, but rather is common problem for Earth-based studies. Testing under continuous microgravity conditions might render different results. This matter could be resolved by testing under real microgravity, for example on the ISS. However, as mentioned, capacity is currently limited. In addition, biohazardous substances and liquids are high risk materials on board the ISS. Future long-term missions to the moon and the construction of the lunar gateway may provide new opportunities^70^.

Furthermore, novel methods to test adherent cells as a key component of biofilm formation could be beneficial in advancing our understanding of this process^71^. The crystal violet assay is an indirect biofilm detection method that measures the extracellular matrix but also total cell biomass of biofilms; hence, it does not distinguishes between live and dead cells^13,72^. Biofilms are complex structures composed of cells and extracellular polymeric substances that can extend beyond just the adherent layer. Crystal violet binds to peptidoglycan in the cell membrane and to EPS components such as proteins, polysaccharides, and extracellular DNA, thereby staining the entire biofilm. This allows for the biofilm being both visible and quantifiable^73^. This semi quantitative method is widely recognized and validated in the scientific community for assessing biofilm formation^74–77^.

Despite its limitations, the crystal violet assay effectively indicates a strain’s ability for increased adherence, which may lead to enhanced biofilm formation capabilities. It is recommended as a screening tool, not a stand-alone experimental tool, which allows for high-throughput screening, making it suitable for our experimental setup^75,78^. A more accurate approach to analyzing enhanced biofilm production would be a quantitative PCR-based assessment or sequencing genes that are hallmarks of biofilm formation. The genomes of several isolates are currently being analyzed to investigate specific genes such as *esp* and other genes involved in biofilm development in Enterococci, including *acm, scm, sgrA*, and *ecbA*. These data will be included in a future publication. Combining both methods, the crystal violet assay together with the analysis of biofilm-related genes, has previously been demonstrated to be successful with Enterococci^72,75^. This highlights the importance of combining phenotypic assays, like the crystal violet assay, with genotypic analysis to obtain a comprehensive view of biofilm formation, which is also effective in multispecies biofilm formation^79^.

In conclusion, we showed that sim. µ*g* can influence antibiotic susceptibility in *E. faecium* isolates, since four isolates decreased and three isolates increased in MIC after sim. µ*g* to seven different antibiotics (Tabel 1). Especially, the MIC increase to vancomycin shows the importance to investigate further how the loss of gravity can change antibiotic susceptibility in *E. faecium*. We showed that two VRE isolates and four VSE isolates exhibited significant changes in adherence under sim. µ*g*, suggesting the potential for increased biofilm formation in these isolates (Figs. 1, 2, 3). These phenotypic changes may arise from changes, involving genomic alterations, or acclimation, involving differential gene expression. Moreover, four VRE, four VSE, and one VVE-B isolate displayed significant changes in their desiccation tolerance after sim. µ*g* (Figs. 4, 5, 6). To further deepen our understanding, especially regarding genetic variability and the selective pressures imposed by the sim. µ*g* environment, it is essential to conduct genotypic analyses as part of future investigations. These analyses will be crucial for uncovering the underlying mechanisms at play.

## Material and methods

### *E. faecium* isolates

In total 42 *E.faecium* isolates (VRE *n* = 15, VVE-B *n* = 7, VSE *n* = 20) from various clinical specimen including three reference strains (DSMZ 17050, ATCC 51559, ATCC 6057) were tested in this study (Table 2).

**Table 2:** Overview of all tested *E. faecium* including the associated vancomycin resistance gene (*vanAB*) determined by PCR, isolation source and specimen

*E. faecium* isolates			
Isolate	*vanAB* gene	Isolation source	Isolation specimen
VRE-1	*vanB*	Clinical isolate	Blood
VRE-2	*vanB*	Clinical isolate	Blood
VRE-3	*vanB*	Clinical isolate	Urine
VRE-4	*vanB*	Clinical isolate	Other clinical specimens
VRE-5	*vanA*	Clinical isolate	Blood
VRE-6	*vanB*	Clinical isolate	Urine
VRE-7	*vanB*	Clinical isolate	Urine
VRE-8	*vanB*	Clinical isolate	Other clinical specimens
VRE-9	*vanB*	Clinical isolate	Other clinical specimens
VRE-10	*vanB*	Clinical isolate	Urine
VRE-11	*vanB*	Clinical isolate	Other clinical specimens
VRE-12	*vanB*	Clinical isolate	Other clinical specimens
VRE-13	*vanB*	Clinical isolate	Blood
ATCC 51559	*vanA*	Clinical isolate	Other clinical specimens
DSMZ 17050	*vanA*	Clinical isolate	Other clinical specimens
VVE-B-16	*vanB*	Clinical isolate	Urine
VVE-B-17	*vanB*	Clinical isolate	Other clinical specimens
VVE-B-18	*vanB*	Clinical isolate	Urine
VVE-B-19	*vanB*	Clinical isolate	Urine
VVE-B-20	*vanB*	Clinical isolate	Other clinical specimens
VVE-B-21	*vanB*	Clinical isolate	Urine
VVE-B-22	*vanB*	Clinical isolate	Blood
VSE-23	/	Clinical isolate	Urine
VSE-24	/	Clinical isolate	Urine
VSE-25	/	Clinical isolate	Urine
VSE-26	/	Clinical isolate	Urine
VSE-27	/	Clinical isolate	Other clinical specimens
VSE-28	/	Clinical isolate	Other clinical specimens
VSE-29	/	Clinical isolate	Other clinical specimens
VSE-30	/	Clinical isolate	Urine
VSE-31	/	Clinical isolate	Other clinical specimens
VSE-32	/	Clinical isolate	Urine
VSE-33	/	Clinical isolate	Urine
VSE-34	/	Clinical isolate	Other clinical specimens
VSE-35	/	Clinical isolate	Other clinical specimens
ATCC 6057	/	Dairy products	Food production
VSE-37	/	Clinical isolate	Other clinical specimens
VSE-38	/	Clinical isolate	Urine
VSE-39	/	Clinical isolate	Urine
VSE-40	/	Clinical isolate	Urine
VSE-39	/	Clinical isolate	Urine
VSE-42	/	Clinical isolate	Other clinical specimens

For this study, 42 *E. faecium* biosafety level 2 isolates, each obtained from distinct patients, including three reference strains (DSMZ 17050, ATCC 51559, ATCC 6057) were tested.

*VRE* vancomycin-resistant *E. faecium* (minimal inhibitory concentration (MIC) >4), *VVE-B* phenotypically tested vancomycin susceptible *E. faecium* isolates harboring a vanB gene (MIC ≤ 4), *VSE* vancomycin susceptible *E. faecium*.

### Cultivation of isolates

For cultivation all *E. faecium* isolates, stored as cryocultures (−80 °C) were streaked out on SBA (sheep blood agar; Oxoid™, Thermo Fisher Scientific Inc., Waltham, MA, USA). The agar plates were incubated at 37 °C for 24 h and stored afterwards at 4 °C. For all experiments, colonies from 24 h old plates were used to set a McFarland standard of 0.5 MFU (McFarland Unit) in NaCl (0.85%) using a nephelometer (DensiCHEK, bioMérieux Inc., USA). The turbidity of 0.5 MFU corresponds approximately to 1.5 × 10^8^ cells/mL^80^.

### Simulation of microgravity

**Fig. 7:**
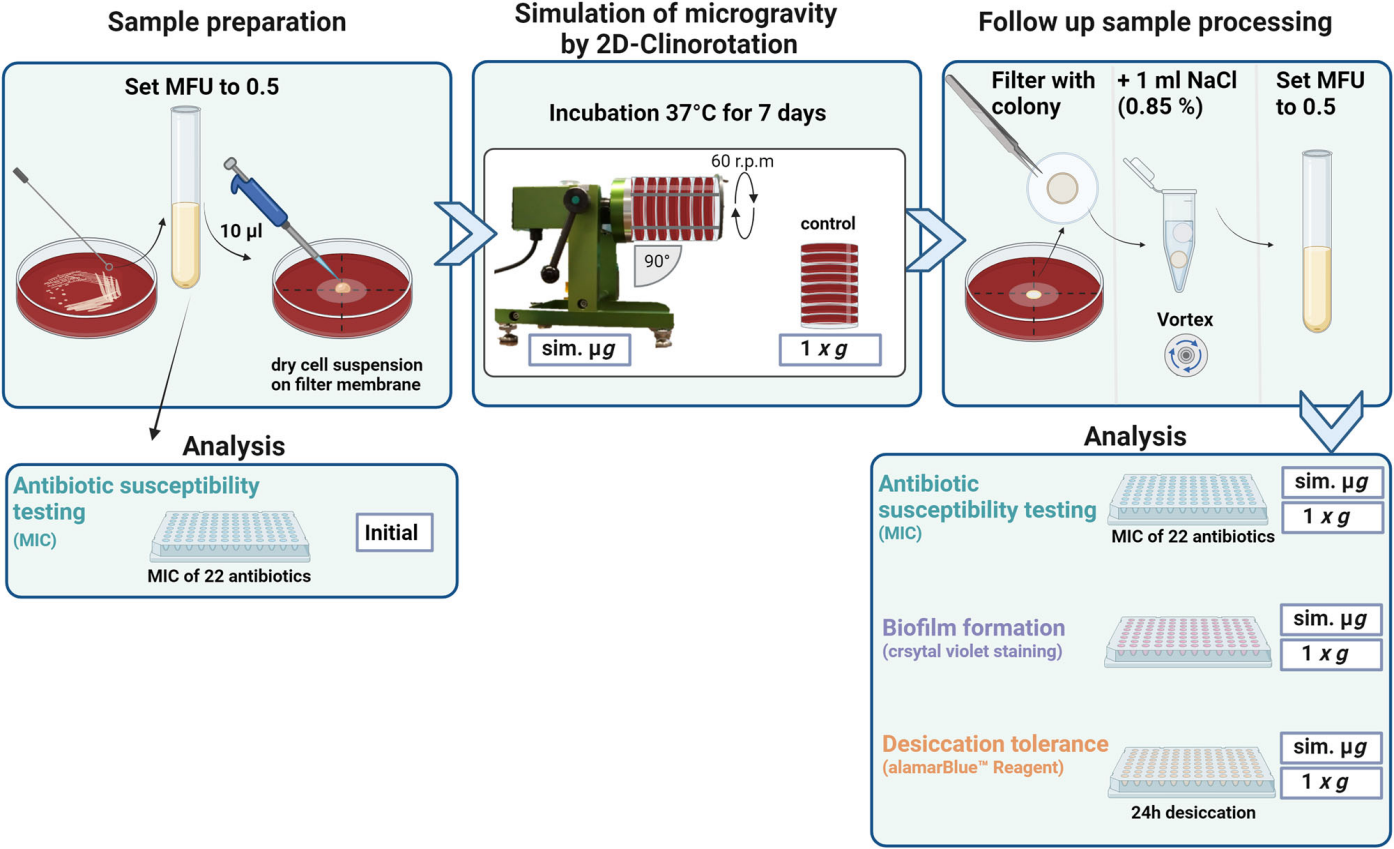
Workflow overview from *E. faecium* sample preparation to final analysis (antibiotic susceptibility testing, biofilm formation, desiccation tolerance) after simulation of microgravity for 7 days by 2D-Clinorotation. MFU McFarland Unit, MIC minimal inhibitory concentration, sim. µ*g* simulated microgravity, NaCl Sodium Chloride 0.85%. Created with BioRender.com.

From the prepared McFarland standard, 10 µL were pipetted on the middle of the IsoporeTM PC membrane filter (0.4 µm, 13 mmØ) (Merck KGaA, Darmstadt, Germany) in the center of a SBA plate. The plates were left under the sterile bench for the droplet to dry. All agar plates were subsequently closed with parafilm. They were then placed into a fast-rotating 2-D clinostat (uniaxial clinostat, UN-KTM2, Advanced Engineering Services, Co. Ltd.) and incubated at 37 °C^81,82^. The rotation axis of the clinostat was aligned parallel to the ground and set to 60 rpm. The controls (1 *g*) were placed next to the clinostat into the incubator (37 °C). After 7 days of incubation, each filter membrane with the grown colony on top was transferred into a 1.5 mL reaction tube with 1 mL NaCl (0.85%). All samples were vortexed for 15 s to dissolve the colony from the membrane filter. A McFarland standard was prepared for further testing of antibiotic susceptibility, biofilm formation and desiccation tolerance (Fig. 7).

### Antibiotic susceptibility testing

To determine the antibiotic susceptibility, standard MIC plates MICRONAUT-S MRSA/GP (Merlin Diagnostika, Bornheim, Germany) were used. The previously described McFarland standard was prepared and 100 µL of the suspension was mixed with 11.5 mL of Mueller Hinton broth. This mixture was applied into the MIC 96-well plate, with 100 µL for each well. After incubation for 18–24 h at 37 °C the plate was evaluated and read visually by analyzing the bacterial growth (visible pinpoint). In total the antibiotic susceptibility for each strain was tested in regads to 22 different antibiotics (Supplementary Material, Table 3, Fig. 8). Each isolate was assessed for susceptibility in triplicates after exposure to both sim. µ*g* and 1 *g* using three MIC plates, with one replicate utilized for the initial testing to determine the MIC of each isolate.

### Desiccation assay

To assess the viability of *E. faecium* cells after 24 h desiccation, the alamarBlue reagent was used. This method enables the detection of metabolic activity by measuring the absorbance (OD_570nm,_ OD_600nm_) with the multi-detection microplate reader (Infinite M1000, Tecan Trading AG, Switzerland). A McFarland standard of each strain was prepared and 100 µL of that suspension were transferred into each well of a 96-well plate in triplicates. As a control, twelve wells were filled with 100 µL NaCl (0.85%) without any cell suspension. The microtiter plate was placed under the sterile bench to dry for 24 h at room temperature. After desiccation (24 h), all wells were mixed with 180 µL of BHI media. Additionally, 20 µL of the alamarBlue™ Cell Viability Reagent (Thermo Fisher Scientific Inc., Waltham, MA, USA) was added. In the multi-detection microplate reader the plate was incubated for 15 h at 37 °C and measurements were taken every 30 min.

### Crystal violet biofilm assay

Biofilm formation was determined according to Stepanovic et al.^76^ with modifications: a McFarland standard for each *E. faecium* strain was prepared. Next, 100 µL of the suspension was pipetted into a 96-well plate and 100 µL of BHI media was added and mixed well. The microtiter plate was wrapped with parafilm and incubated for 24 h at 37 °C. Afterwards, the cell suspension was discarded and all used wells were carefully washed twice with 200 µL PBS. The microtiter plate was left to dry for 10 min. Then 100 µL 0.5% crystal violet (Merck KGaA, Darmstadt, Germany) was added and the plate was covered with aluminum foil for 30 min at room temperature. Next the supernatant was discarded followed by three washing stepswith 200 µL distilled water, respectively. Last, each well was filled with 300 µL EtOH (≥99.8%) and put on a shaker at room temperature for 5 min to dissolve the staining from the biofilm. The optical density (OD_600nm_) measurement of the biofilm in each well of the 96-well plate was done with the multi-detection microplate reader.

### Data analysis

All figures and statistical analysis were processed with Excel (Microsoft Office Standard 2019) and SigmaPlot (Systat Software, Version 14.5). If sampling size was applicable for statistical analysis the two-sample Student’s *t*-test was performed. The presented data are depicted by the arithmetic means with the corresponding with calculated standard error.

## Supplementary information

Supplementary Information

## Data Availability

The datasets used and analyzed during the current study are available from the corresponding author on reasonable request.
