# Supplementary material for: Systematic screening of 42 vancomycin-resistant Enterococcus faecium strains for resistance, biofilm, and desiccation in simulated microgravity
Source: NPJ Microgravity. 2024 Nov 13;10:103. doi: 10.1038/s41526-024-00447-8 (PMC11561132; doi:10.1038/s41526-024-00447-8)
Supplement: Supplementary file 1 — Supplementary Information [file 41526_2024_447_MOESM1_ESM.pdf]

Supplementary information

Supplementary Table 1 Changes of antibiotic susceptibility after sim. µ*g* and/or 1 *g* in *E. faecium* isolates. All MIC changes in this table are equal to or less than one step. Alterations are displayed in minimal inhibitory concentration MIC (µg/mL) for each tested antibiotic. Initial: initial MIC of isolate, sim. µ*g*: MIC after incubation under sim. µ*g* for 7 days, 1 *g* (control): MIC after incubation for 7 days, Isolate: VRE (vancomycin resistant Enterococci), VVE-B (vancomycin variable Enterococci VanB type), VSE (vancomycin susceptible Enterococci). All isolates tested in triplicates (except for VRE-8 and VRE-13, which were excluded due to contamination, these isolates were tested in duplicates).

One step changes of MIC (µg/mL)

Isolate	Initial	Sim. µ*g*	1 *g*	Antibiotic
ATCC 51559	<1	2	2	linezolid
ATCC 51559	1	<0,5	<0,5	quinupristin/ dalfopristin
DSMZ 17050	<1	>2	<1	fusidic acid
DSMZ 17050	<1	256	256	mupirocin
DSMZ 17050	1	2	2	quinupristin/ dalfopristin
DSMZ 17050	0,5/ 9,5	0,03125/ 0,59375	0,03125/ 0,59375	trimethoprim/ sulfamethoxazole
VRE-1	32	64	64	fosfomycin
VRE-1	0,5	1	1	teicoplanin
VRE-1	0,03125/ 0,59375	0,03125/ 0,59375	0,5/ 9,5	trimethoprim/ sulfamethoxazole
VRE-2	2	4	4	daptomycin
VRE-2	>1	256	256	mupirocin
VRE-2	1	>2	>2	rifampicin
VRE-4	2	4	4	daptomycin
VRE-4	0,5	1	1	teicoplanin
VRE-5	1	2	2	daptomycin
VRE-5	1	>2	>2	fusidic acid
VRE-5	4	2	2	gentamycin
VRE-7	2	<1	<1	linezolid
VRE-8	2	<1	<1	linezolid
VRE-9	1	2	2	daptomycin
VRE-9	<1	256	256	mupirocin
VRE-11	1	2	2	gentamycin
VRE-11	<1	256	256	mupirocin
VRE-13	<1	256	256	mupirocin
ATCC 6057	32	64	32	fosfomycin
ATCC 6057	<1	>2	<1	fusidic acid
ATCC 6057	<1	256	256	mupirocin
VSE-23	>1	256	256	mupirocin
VSE-24	2	4	4	synercid
VSE-25	4	2	2	gentamycin
VSE-25	1	1	<0,5	quinupristin/ dalfopristin
VSE-27	1	256	256	mupirocin
VSE-28	1	256	256	mupirocin
VSE-29	<1	256	256	upirocin

VSE-30	>16	>16	>8	ampicillin
VSE-30	2	2	4	gentamycin
VSE-30	2	<1	2	linezolid
VSE-30	0,5	1	0,5	vancomycin
VSE-31	2	4	4	quinupristin/ dalfopristin
VSE-32	0,5	0,5	1	vancomycin
VSE-33	<1	2	2	linezolid
VSE-34	>8	4	>8	gentamycin
VSE-34	1	1	2	quinupristin/ dalfopristin
VSE-35	<1	>2	>2	fusidic acid
VSE-35	1	1	2	synercid
VSE-37	0,03125/ 0,59375	0,5/ 9,5	<0,03125/ 0,59375	trimethoprim/ sulfamethoxazole
VSE-38	<128	<128	>500	gentamycin high level
VSE-38	2	1	1	vancomycin
VSE-39	2	4	4	daptomycin
VSE-39	<1	256	256	mupirocin
VSE-39	2	4	4	quinupristin/ dalfopristin
VSE-39	0,5	1	1	teicoplanin
VSE-40	<1	256	256	mupirocin
VSE-40	0,5	0,5	1	vancomycin
VSE-41	4	4	2	gentamycin
VSE-41	<0,5	1	1	quinupristin/ dalfopristin
VSE-42	2	<1	<1	linezolid
VSE-42	1	256	256	mupirocin
VSE-42	0,03125/ 0,59375	0,5/ 9,5	0,03125/ 0,59375	trimethoprim/ sulfamethoxazole
VSE-42	0,5	1	1	vancomycin
VVE-B-16	2	4	4	daptomycin
VVE-B-16	1	2	2	gentamycin
VVE-B-17	2	4	4	daptomycin
VVE-B-17	2	2	4	gentamycin
VVE-B-17	>1	256	256	mupirocin
VVE-B-17	<0,5	1	<0,5	quinupristin/ dalfopristin
VVE-B-17	0,03125/ 0,59375	0,5/ 9,5	0,5/ 9,5	trimethoprim/ sulfamethoxazole
VVE-B-18	2	4	4	daptomycin
VVE-B-18	2	<1	<1	linezolid
VVE-B-18	0,5	0,5	1	teicoplanin
VVE-B-19	<1	256	256	mupirocin
VVE-B-20	1	2	2	gentamycin
VVE-B-20	<1	256	256	mupirocin
VVE-B-21	0,5	0,5	1	teicoplanin

Supplementary Table 2 Changes of antibiotic susceptibility after sim. µ*g* and/or 1 *g* in *E. faecium* isolates. All MIC changes in this table are more than one step changes, identical in MIC after incubation in sim. µ*g* and in 1 *g* control (incubation only). Alterations are displayed in minimal inhibitory concentration MIC (µg/mL) for each tested antibiotic. Initial: initial MIC of isolate, sim. µ*g*: MIC after incubation under sim. µ*g* for 7 days, 1 *g* (control): MIC after incubation for 7 days, Isolate: VRE (vancomycin Resistant Enterococci), VVE-B (vancomycin variable Enterococci VanB type), VSE (vancomycin susceptible Enterococci). All isolates tested in triplicates (except for VRE-8 and VRE-10, which were excluded due to contamination, these isolates were tested in duplicates)

More than one step changes of MIC (µg/mL)

Isolate	Initial	Sim. µ*g*	1 *g*	Antibiotic
DSMZ 17050	<8	64	64	fosfomycin
VRE-1	>4	<0,5	<0,5	quinupristin/ dalfopristin
VRE-3	<0,5/ 9,5	>4/ 76	>4/ 76	trimethoprim/ sulfamethoxazole
VRE-8	0,5	>2	>2	rifampicin
VRE-9	0,5/ 9,5	>4/ 76	>4/ 76	trimethoprim/ sulfamethoxazole
VRE-10	0,5	>2	>2	rifampicin
VSE-25	>16	<2	<2	ampicillin
VSE-26	>16	<2	<2	ampicillin
VSE-26	>8	2	2	penicillin G
VSE-26	<0,5	>4	>4	quinupristin/ dalfopristin
VSE-27	0,03125/ 0,59375	>4/ 76	>4/ 76	trimethoprim/ sulfamethoxazole
VSE-35	>16	<2	<2	ampicillin
VSE-35	>8	2	2	penicillin G
VSE-35	8	0,5	0,5	vancomycin
VVE-B-16	<0,25	>4	>4	erythromycin
VVE-B-19	2	32	32	vancomycin
VVE-B-20	<0,5	2	2	daptomycin
VVE-B-20	8	32	32	vancomycin

Supplementary Table 3 List of antibiotics and their according concentrations (µg/mL) of MIC 96-well plates (MICRONAUT-S MRSA/GP, MERLIN) for susceptibility testing by broth microdilution. All 22 different antibiotics were tested in this study for each isolate in triplicates.

Nr.	Abbreviation	Antibiotic	MIC (µg/mL)
1	PEN	penicillin G	0.0625-8
2	AMP	ampicillin	2-16
3	CFL	ceftarolin	0.25-2
4	OXA	oxacillin	0.125-16
5	RAM	rifampicin	0.0625-2
6	COX	cefoxitin	2-16
7	TPL	teicoplanin	0.125-16
8	VAN	vancomycin	0.25-32
9	FOS	fosfomycin	8-64
10	SYN	quinupristin/ dalfopristin (Synercid)	0.5-4
11	LIZ	linezolid	1-8
12	DPT	daptomycin	0.5-4
13	TGC	tigecyline	0.125-1
14	MOX	moxifloxacin	0.25-2
15	GNH	gentamycin high level	128-500
16	MUP	mupirocin	1-256
17	T/S	trimethoprim/sulfamethoxazole	0.03125/0.59375
18	ERC	erythromycin/clindamycin	4/0.5
19	CLI	clindamycin	0.5
20	FUS	fusidic acid	1-2
21	GEN	gentamycin	0.5-8
22	ERY	erythromycin	0.25-4

Supplementary Figure 8 Layout of MIC 96-well plate (MICRONAUT-S MRSA/GP, MERLIN) for susceptibility testing by broth microdilution. In each well the abbreviation of the antibiotic and the according concentration (µg/mL) is listed. GC: growth control.

	1	2	3	4	5	6	7	8	9	10	11	12
**A**	PEN 8	AMP 16	OXA 16	RAM 2	TPL 16	VAN 32	FOS 64	LIZ 8	TGC 1	GNH 128	GNH 500	MUP 256
**B**	PEN 4	AMP 8	OXA 8	RAM 1	TPL 8	VAN 16	FOS 32	LIZ 4	TGC 0.5	T/S 4/76	FUS 2	MUP 1
**C**	PEN 2	AMP 4	OXA 4	RAM 0.5	TPL 4	VAN 8	FOS 16	LIZ 2	TGC 0.25	T/S 2/38	FUS 1	ERY 4
**D**	PEN 1	AMP 2	OXA 2	RAM 0.0625	TPL 2	VAN 4	FOS 8	LIZ 1	TGC 0.125	T/S 1/19	GEN 8	ERY 2
**E**	PEN 0.5	CFL 2	OXA 1	COX 16	TPL 1	VAN 2	SYN 4	DPT 4	MOX 2	T/S 0.5/9.5	GEN 4	ERY 1
**F**	PEN 0.25	CFL 1	OXA 0.5	COX 8	TPL 0.5	VAN 1	SYN 2	DPT 2	MOX 1	T/S 0.03125/ 0.59375	GEN 2	ERY 0.5
**G**	PEN 0.125	CFL 0.5	OXA 0.25	COX 4	TPL 0.25	VAN 0.5	SYN 1	DPT 1	MOX 0.5	ERC 4/0.5	GEN 1	ERY 0.25
**H**	PEN 0.0625	CFL 0.25	OXA 0.125	COX 2	TPL 0.125	VAN 0.25	SYN 0.5	DPT 0.5	MOX 0.25	CLI 0.5	GEN 0.5	GC

Supplementary Table 4 Changes in antibiotic susceptibility after sim. µ*g* and 1 *g* of *E. faecium* Isolates (n = 42). Alterations displayed in MIC (µg/mL) for each tested antibiotic (n = 22). Initial: MIC test of isolate, sim. µ*g*: MIC test after 7 days of incubation under sim. µ*g* using a 2D-Clinostat, 1 *g* (control): MIC test after 7 days of incubation under standard gravity, Isolate: VRE (vancomycin resistant *E. faecium*), VVE-B (vancomycin variable *E. faecium* VanB type), VSE (vancomycin susceptible *E. faecium*). For the initial MIC testing one replicate (Rep. 1) was performed to determine the MIC values. For each condition (sim. µ*g*, 1 *g)* three replicates were tested (Rep. 1-3). Data from replicates that showed different results within the three replicates (Rep. 1-3) were excluded from our analysis to ensure consistency and reliability.

VRE-1

	Intital	1 *g*			sim. µ*g*		
Antibiotic	Rep. 1	Rep. 1	Rep. 2	Rep. 3	Rep. 1	Rep. 2	Rep. 3
Penicillin G	>8	>8			>8		
Ampicillin	>16	>16			>16		
Ceftarolin	>2	>2			>2		
Oxacillin	>16	>16			>16		
Rifampicin	>2	>2			>2		
Cefoxitin	>16	>16			>16		
Teicoplanin	0.5	1			1		
Vancomycin	>32	32			32		
Fosfomycin	32	64			64		
Synercid	>4	<0.5			<0.5		
Linezolid	1	<1			<1		
Daptomycin	2	2	4	4	4		
Tigecyline	<0.125	<0.125			<0.125		
Moxifloxacin	>2	>2			2		
Gentamycin High level	<128	<128			<128		
Mupirocin	256	256			256		
Trimethoprim/Sulfamethoxazole	0.03125/0.59375	0.5/9.5			0.03125/0.59375		
Erythromycin/Clindamycin	4/0.5	4/0.5			4/0.5		
Clindamycin	0.5	0.5			0.5		
Fusidic acid	2	2			>2		
Gentamycin	2	2			2	2	4
Erythromycin	4	4			4		

VRE-2

	Initial	1 *g*			sim. µ*g*		
Antibiotic	Rep. 1	Rep. 1	Rep. 2	Rep. 3	Rep. 1	Rep. 2	Rep. 3
Penicillin G	>8	>8			>8		
Ampicillin	>16	>16			>16		
Ceftarolin	>2	>2			>2		
Oxacillin	>16	>16			>16		
Rifampicin	1	>2			>2		
Cefoxitin	>16	>16			>16		
Teicoplanin	0.5	0.5			0.5		
Vancomycin	>32	32			32		
Fosfomycin	>64	64			64		
Synercid	<0.5	<0.5			<0.5		
Linezolid	<1	<1			<1		
Daptomycin	2	4			4		
Tigecyline	<0.125	<0.125			<0.125		
Moxifloxacin	>2	>2			2		
Gentamycin High level	<128	<128			<128		
Mupirocin	>1	256			256		
Trimethoprim/Sulfamethoxazole	0.03125/0.59375	0.03125/0.59375			0.03125/0.59375		
Erythromycin/Clindamycin	4/0.5	4/0.5			4/0.5		
Clindamycin	0.5	0.5			0.5		
Fusidic acid	2	2			>2		
Gentamycin	1	1			4		
Erythromycin	4	4			4		

VRE-3

	Initial	1 *g*			sim. µ*g*		
Antibiotic	Rep. 1	Rep. 1	Rep. 2	Rep. 3	Rep. 1	Rep. 2	Rep. 3
Penicillin G	>8	>8			>8		
Ampicillin	>16	>16			>16		
Ceftarolin	>2	>2			>2		
Oxacillin	>16	>16			>16		
Rifampicin	>2	>2			>2		
Cefoxitin	>16	>16			>16		
Teicoplanin	0.5	0.5			0.5		
Vancomycin	>32	>32			>32		
Fosfomycin	>64	>64			>64		
Synercid	<0.5	4	<0.5	2	<0.5		
Linezolid	<1	<1			<1		
Daptomycin	2	2			2		
Tigecyline	<0.125	<0.125			<0.125		
Moxifloxacin	>2	>2			>2		
Gentamycin High level	<128	<128			<128		
Mupirocin	256	256			256		
Trimethoprim/Sulfamethoxazole	<0.5/9.5	>4/76			>4/76		
Erythromycin/Clindamycin	>4/0.5	>4/0.5			>4/0.5		
Clindamycin	>0.5	>0.5			>0.5		
Fusidic acid	>2	>2			>2		
Gentamycin	2	2	8	8	2		
Erythromycin	>4	>4			>4		

VRE-4

	Initial	1 *g*			sim. µ*g*		
Antibiotic	Rep. 1	Rep. 1	Rep. 2	Rep. 3	Rep. 1	Rep. 2	Rep. 3
Penicillin G	>8	>8			>8		
Ampicillin	>16	>16			>16		
Ceftarolin	>2	>2			>2		
Oxacillin	>16	>16			>16		
Rifampicin	>2	>2			>2		
Cefoxitin	>16	>16			>16		
Teicoplanin	0.5	1			1		
Vancomycin	>32	>32			>32		
Fosfomycin	64	>64	>64	64	>64		
Synercid	1	>4			<0.5		
Linezolid	<1	<1			<1		
Daptomycin	2	4			4		
Tigecyline	<0.125	<0.125			<0.125		
Moxifloxacin	>2	>2			>2		
Gentamycin High level	<128	<128			<128		
Mupirocin	256	256			256		
Trimethoprim/Sulfamethoxazole	0.03125/0.59375	0.03125/0.59375			0.03125/0.59375		
Erythromycin/Clindamycin	>4/0.5	>4/0.5			>4/0.5		
Clindamycin	>0.5	>0.5			>0.5		
Fusidic acid	>2	>2			>2		
Gentamycin	2	2	1	1	2		
Erythromycin	>4	>4			>4		

VRE-5

	**Initial**	**1 *g***			**sim. µ*g***		
Antibiotic	Rep. 1	Rep. 1	Rep. 2	Rep. 3	Rep. 1	Rep. 2	Rep. 3
Penicillin G	>8		>8			>8	
Ampicillin	>16		>16			>16	
Ceftarolin	>2		>2			>2	
Oxacillin	>16		>16			>16	
Rifampicin	>2		>2			>2	
Cefoxitin	>16		>16			>16	
Teicoplanin	>16		>16			>16	
Vancomycin	>32		>32			>32	
Fosfomycin	>64		>64			>64	
Synercid	1		1			1	
Linezolid	<1		<1			<1	
Daptomycin	1		2			2	
Tigecyline	<0.125		<0.125			<0.125	
Moxifloxacin	>2		>2			>2	
Gentamycin High level	<128		<128			<128	
Mupirocin	256		256			256	
Trimethoprim/Sulfamethoxazole	0.03125/0.59375		0.03125/0.59375			0.03125/0.59375	
Erythromycin/Clindamycin	>4/0.5		>4/0.5			>4/0.5	
Clindamycin	>0.5		>0.5			>0.5	
Fusidic acid	1		>2			>2	
Gentamycin	4		2			2	
Erythromycin	>4		>4			>4	

VRE-6

<table>
<tr><th></th><th>Initial</th><th colspan="2">1 g</th><th colspan="3">sim. μg</th></tr>
<tr><th>Antibiotic</th><th>Rep. 1</th><th>Rep. 1</th><th>Rep. 2</th><th>Rep. 1</th><th>Rep. 2</th><th>Rep. 3</th></tr>
<tr><td>Penicillin G</td><td>>8</td><td colspan="2">>8</td><td colspan="3">>8</td></tr>
<tr><td>Ampicillin</td><td>>16</td><td colspan="2">>16</td><td colspan="3">>16</td></tr>
<tr><td>Ceftarolin</td><td>>2</td><td colspan="2">>2</td><td colspan="3">>2</td></tr>
<tr><td>Oxacillin</td><td>>16</td><td colspan="2">>16</td><td colspan="3">>16</td></tr>
<tr><td>Rifampicin</td><td>0.5</td><td colspan="2">>2</td><td>>2</td><td>0.5</td><td>0.5</td></tr>
<tr><td>Cefoxitin</td><td>>16</td><td colspan="2">>16</td><td colspan="3">>16</td></tr>
<tr><td>Teicoplanin</td><td>0.25</td><td colspan="2">>16</td><td colspan="3">0.25</td></tr>
<tr><td>Vancomycin</td><td>>32</td><td colspan="2">>32</td><td colspan="3">>32</td></tr>
<tr><td>Fosfomycin</td><td>>64</td><td colspan="2">>64</td><td colspan="3">>64</td></tr>
<tr><td>Synercid</td><td>0.5</td><td colspan="2">0.5</td><td>0.5</td><td>1</td><td>1</td></tr>
<tr><td>Linezolid</td><td><1</td><td colspan="2"><1</td><td colspan="3"><1</td></tr>
<tr><td>Daptomycin</td><td>2</td><td>2</td><td>4</td><td>4</td><td>2</td><td>2</td></tr>
<tr><td>Tigecyline</td><td><0.125</td><td colspan="2"><0.125</td><td colspan="3"><0.125</td></tr>
<tr><td>Moxifloxacin</td><td>>2</td><td colspan="2">>2</td><td colspan="3">>2</td></tr>
<tr><td>Gentamycin High level</td><td><128</td><td colspan="2"><128</td><td colspan="3"><128</td></tr>
<tr><td>Mupirocin</td><td>256</td><td colspan="2">256</td><td colspan="3">256</td></tr>
<tr><td>Trimethoprim/Sulfamethoxazole</td><td>0.03125/0.59375</td><td colspan="2">0.03125/0.59375</td><td colspan="3">0.03125/0.59375</td></tr>
<tr><td>Erythromycin/Clindamycin</td><td>>4/0.5</td><td colspan="2">>4/0.5</td><td colspan="3">>4/0.5</td></tr>
<tr><td>Clindamycin</td><td>>0.5</td><td colspan="2">>0.5</td><td colspan="3">>0.5</td></tr>
<tr><td>Fusidic acid</td><td>>2</td><td colspan="2">>2</td><td><1</td><td>2</td><td>>2</td></tr>
<tr><td>Gentamycin</td><td>8</td><td>4</td><td>8</td><td>4</td><td>8</td><td>4</td></tr>
<tr><td>Erythromycin</td><td>>4</td><td colspan="2">>4</td><td colspan="3">>4</td></tr>
</table>

VRE-7

<table>
<tr><th></th><th>Initial</th><th colspan="3">1 g</th><th colspan="3">sim. μg</th></tr>
<tr><th>Antibiotic</th><th>Rep. 1</th><th>Rep. 1</th><th>Rep. 2</th><th>Rep. 3</th><th>Rep. 1</th><th>Rep. 2</th><th>Rep. 3</th></tr>
<tr><td>Penicillin G</td><td>>8</td><td colspan="3">>8</td><td colspan="3">>8</td></tr>
<tr><td>Ampicillin</td><td>>16</td><td colspan="3">>16</td><td colspan="3">>16</td></tr>
<tr><td>Ceftarolin</td><td>>2</td><td colspan="3">>2</td><td colspan="3">>2</td></tr>
<tr><td>Oxacillin</td><td>>16</td><td colspan="3">>16</td><td colspan="3">>16</td></tr>
<tr><td>Rifampicin</td><td>>2</td><td colspan="3">>2</td><td colspan="3">>2</td></tr>
<tr><td>Cefoxitin</td><td>>16</td><td colspan="3">>16</td><td colspan="3">>16</td></tr>
<tr><td>Teicoplanin</td><td>0.5</td><td colspan="3">0.5</td><td colspan="3">0.5</td></tr>
<tr><td>Vancomycin</td><td>>32</td><td colspan="3">>32</td><td colspan="3">>32</td></tr>
<tr><td>Fosfomycin</td><td>64</td><td colspan="3">64</td><td colspan="3">>64</td></tr>
<tr><td>Synercid</td><td><0.5</td><td><0.5</td><td><0.5</td><td>1</td><td colspan="3"><0.5</td></tr>
<tr><td>Linezolid</td><td>2</td><td colspan="3"><1</td><td colspan="3"><1</td></tr>
<tr><td>Daptomycin</td><td>1</td><td>1</td><td>1</td><td>2</td><td colspan="3">1</td></tr>
<tr><td>Tigecyline</td><td><0.125</td><td colspan="3"><0.125</td><td colspan="3"><0.125</td></tr>
<tr><td>Moxifloxacin</td><td>>2</td><td colspan="3">>2</td><td colspan="3">>2</td></tr>
<tr><td>Gentamycin High level</td><td><128</td><td colspan="3"><128</td><td colspan="3"><128</td></tr>
<tr><td>Mupirocin</td><td>256</td><td colspan="3">256</td><td colspan="3">256</td></tr>
<tr><td>Trimethoprim/Sulfamethoxazole</td><td>0.03125/0.59375</td><td colspan="3">0.03125/0.59375</td><td colspan="3">0.03125/0.59375</td></tr>
<tr><td>Erythromycin/Clindamycin</td><td>>4/0.5</td><td colspan="3">>4/0.5</td><td colspan="3">>4/0.5</td></tr>
<tr><td>Clindamycin</td><td>>0.5</td><td colspan="3">>0.5</td><td colspan="3">>0.5</td></tr>
<tr><td>Fusidic acid</td><td>>2</td><td colspan="3">>2</td><td colspan="3">>2</td></tr>
<tr><td>Gentamycin</td><td>1</td><td colspan="3">1</td><td colspan="3">1</td></tr>
<tr><td>Erythromycin</td><td>>4</td><td colspan="3">>4</td><td colspan="3">>4</td></tr>
</table>

VRE-8

	Initial	1 *g*		sim. µ*g*		
Antibiotic	Rep. 1	Rep. 1	Rep. 2	Rep. 1	Rep. 2	Rep. 3
Penicillin G	>8	>8		>8		
Ampicillin	>16	>16		>16		
Ceftarolin	>2	>2		>2		
Oxacillin	>16	>16		>16		
Rifampicin	0.5	>2		>2		
Cefoxitin	>16	>16		>16		
Teicoplanin	0.5	0.5		0.5		
Vancomycin	>32	>32		>32		
Fosfomycin	64	>64		64		
Synercid	<0.5	<0.5		<0.5		
Linezolid	2	<1		<1		
Daptomycin	2	2		2		
Tigecyline	<0.125	<0.125		<0.125		
Moxifloxacin	>2	>2		>2		
Gentamycin High level	<128	<128		<128		
Mupirocin	256	256		256		
Trimethoprim/Sulfamethoxazole	0.03125/0.59375	0.03125/0.59375		0.03125/0.59375		
Erythromycin/Clindamycin	>4/0.5	>4/0.5		>4/0.5		
Clindamycin	>0.5	>0.5		>0.5		
Fusidic acid	>2	>2		>2		
Gentamycin	2	2	1	1	<0.5	1
Erythromycin	>4	>4		>4		

VRE-9

	Initial	1 *g*			sim. µ*g*		
Antibiotic	Rep. 1	Rep. 1	Rep. 2	Rep. 3	Rep. 1	Rep. 2	Rep. 3
Penicillin G	>8	>8			>8		
Ampicillin	>16	>16			>16		
Ceftarolin	>2	>2			>2		
Oxacillin	>16	>16			>16		
Rifampicin	>2	>2			>2		
Cefoxitin	>16	>16			>16		
Teicoplanin	0.25	0.5			0.5		
Vancomycin	32	>32			>32		
Fosfomycin	32	64			64	>64	>64
Synercid	2	>4			>4	>4	4
Linezolid	<1	<1			<1		
Daptomycin	1	2			2		
Tigecyline	<0.125	<0.125			<0.125		
Moxifloxacin	>2	>2			>2		
Gentamycin High level	<128	<128			<128		
Mupirocin	<1	256			256		
Trimethoprim/Sulfamethoxazole	0.5/9.5	>4/76			>4/76		
Erythromycin/Clindamycin	>4/0.5	>4/0.5			>4/0.5		
Clindamycin	>0.5	>0.5			>0.5		
Fusidic acid	>2	>2			>2		
Gentamycin	2	2			2		
Erythromycin	>4	>4			>4		

VRE-10

<table>
<tr><th></th><th>Initial</th><th colspan="2">1 g</th><th colspan="2">sim. µg</th></tr>
<tr><th>Antibiotic</th><th>Rep. 1</th><th>Rep. 1</th><th>Rep. 2</th><th>Rep. 1</th><th>Rep. 2</th></tr>
<tr><td>Penicillin G</td><td>>8</td><td colspan="2">>8</td><td colspan="2">>8</td></tr>
<tr><td>Ampicillin</td><td>>16</td><td colspan="2">>16</td><td colspan="2">>16</td></tr>
<tr><td>Ceftarolin</td><td>>2</td><td colspan="2">>2</td><td colspan="2">>2</td></tr>
<tr><td>Oxacillin</td><td>>16</td><td colspan="2">>16</td><td colspan="2">>16</td></tr>
<tr><td>Rifampicin</td><td>0.5</td><td colspan="2">>2</td><td colspan="2">>2</td></tr>
<tr><td>Cefoxitin</td><td>>16</td><td colspan="2">>16</td><td colspan="2">>16</td></tr>
<tr><td>Teicoplanin</td><td>0.5</td><td colspan="2">0.5</td><td>1</td><td>0.5</td></tr>
<tr><td>Vancomycin</td><td>32</td><td colspan="2">>32</td><td colspan="2">>32</td></tr>
<tr><td>Fosfomycin</td><td>64</td><td>>64</td><td>64</td><td colspan="2">>64</td></tr>
<tr><td>Synercid</td><td><0.5</td><td colspan="2"><0.5</td><td colspan="2"><0.5</td></tr>
<tr><td>Linezolid</td><td>4</td><td colspan="2">4</td><td>2</td><td>4</td></tr>
<tr><td>Daptomycin</td><td>1</td><td colspan="2">2</td><td colspan="2">4</td></tr>
<tr><td>Tigecyline</td><td><0.125</td><td colspan="2"><0.125</td><td colspan="2"><0.125</td></tr>
<tr><td>Moxifloxacin</td><td>>2</td><td colspan="2">>2</td><td colspan="2">>2</td></tr>
<tr><td>Gentamycin High level</td><td><128</td><td colspan="2"><128</td><td colspan="2"><128</td></tr>
<tr><td>Mupirocin</td><td>256</td><td colspan="2">256</td><td colspan="2">256</td></tr>
<tr><td>Trimethoprim/Sulfamethoxazole</td><td>0.5/9.5</td><td>0.03125/0.59375</td><td>0.5/9.5</td><td>0.5/9.5</td><td>1/19</td></tr>
<tr><td>Erythromycin/Clindamycin</td><td>>4/0.5</td><td colspan="2">>4/0.5</td><td colspan="2">>4/0.5</td></tr>
<tr><td>Clindamycin</td><td>>0.5</td><td colspan="2">>0.5</td><td colspan="2">>0.5</td></tr>
<tr><td>Fusidic acid</td><td>>2</td><td colspan="2">>2</td><td colspan="2">>2</td></tr>
<tr><td>Gentamycin</td><td>2</td><td colspan="2">2</td><td colspan="2">2</td></tr>
<tr><td>Erythromycin</td><td>>4</td><td colspan="2">>4</td><td colspan="2">>4</td></tr>
</table>

VRE-11

	Initial	1 *g*			sim. µ*g*		
Antibiotic	Rep. 1	Rep. 1	Rep. 2	Rep. 3	Rep. 1	Rep. 2	Rep. 3
Penicillin G	>8	>8			>8		
Ampicillin	>16	>16			>16		
Ceftarolin	>2	>2			>2		
Oxacillin	>16	>16			>16		
Rifampicin	>2	>2			>2		
Cefoxitin	>16	>16			>16		
Teicoplanin	0.25	0.5			1	4	0.5
Vancomycin	>32	>32			>32		
Fosfomycin	64	>64	64	>64	>64	64	64
Synercid	<0.5	1			2	1	1
Linezolid	2	<1			<1	<1	2
Daptomycin	2	4			4	2	4
Tigecyline	<0.125	<0.125			<0.125		
Moxifloxacin	>2	>2			>2		
Gentamycin High level	<128	<128			<128		
Mupirocin	<1	256			256		
Trimethoprim/ Sulfamethoxazole	0.5/ 9.5	0.03125/ 0.59375	0.5/ 9.5	0.5/ 9.5	0.5/ 9.5	0.03125/ 0.59375	0.5/ 9.5
Erythromycin/Clindamycin	>4/0.5	>4/0.5			>4/0.5		
Clindamycin	>0.5	>0.5			>0.5		
Fusidic acid	>2	>2			>2		
Gentamycin	1	2			2		
Erythromycin	>4	>4			>4		

VRE-12

	Initial	1 *g*			sim. µ*g*		
Antibiotic	Rep. 1	Rep. 1	Rep. 2	Rep. 3	Rep. 1	Rep. 2	Rep. 3
Penicillin G	>8	>8			>8		
Ampicillin	>16	>16			>16		
Ceftarolin	>2	>2			>2		
Oxacillin	>16	>16			>16		
Rifampicin	>2	>2			>2		
Cefoxitin	>16	>16			>16		
Teicoplanin	0.5	0.5			1	1	0.5
Vancomycin	32	32			32		
Fosfomycin	64	64			64		
Synercid	1	1			1		
Linezolid	2	2			2		
Daptomycin	4	4			>4	4	>4
Tigecyline	<0.125	<0.125			<0.125		
Moxifloxacin	>2	>2			>2		
Gentamycin High level	<128	<128			<128		
Mupirocin	256	256			256		
Trimethoprim/Sulfamethoxazole	0.5/9.5	0.5/9.5			>4/76		
Erythromycin/Clindamycin	>4/0.5	>4/0.5			>4/0.5		
Clindamycin	>0.5	>0.5			>0.5		
Fusidic acid	>2	>2			>2		
Gentamycin	2	2			2		
Erythromycin	>4	>4			>4		

VRE-13

	Initial	1 *g*		sim. µ*g*		
Antibiotic	Rep. 1	Rep. 1	Rep. 2	Rep. 1	Rep. 2	Rep. 3
Penicillin G	>8	>8		>8		
Ampicillin	>16	>16		>16		
Ceftarolin	>2	>2		>2		
Oxacillin	>16	>16		>16		
Rifampicin	>2	>2		>2		
Cefoxitin	>16	>16		>16		
Teicoplanin	0.5	2	0.5	>16	0.5	
Vancomycin	32	>32		32	>32	
Fosfomycin	64	>64		>64		
Synercid	1	<0.5		1	<0.5	1
Linezolid	<1	<1		<1		
Daptomycin	2	4	2	4	2	2
Tigecyline	<0.125	<0.125		<0.125		
Moxifloxacin	>2	>2		>2		
Gentamycin High level	<128	<128		<128		
Mupirocin	<1	256		256		
Trimethoprim/Sulfamethoxazole	0.03125/0.59375	0.03125/0.59375		0.03125/0.59375		
Erythromycin/Clindamycin	>4/0.5	>4/0.5		>4/0.5		
Clindamycin	>0.5	>0.5		>0.5		
Fusidic acid	>2	>2		>2		
Gentamycin	2	2	1	4	2	1
Erythromycin	>4	>4		>4		

ATCC 51559

Antibiotic	Initial	1 *g*			sim. µ*g*		
	Rep. 1	Rep. 1	Rep. 2	Rep. 3	Rep. 1	Rep. 2	Rep. 3
Penicillin G	>8	>8			>8		
Ampicillin	>16	>16			>16		
Ceftarolin	>2	>2			>2		
Oxacillin	>16	>16			>16		
Rifampicin	2	>2			>2		
Cefoxitin	>16	>16			>16		
Teicoplanin	>16	>16			>16		
Vancomycin	>32	>32			>32		
Fosfomycin	>64	>64			>64		
Synercid	1	<0.5			<0.5		
Linezolid	<1	2			2		
Daptomycin	2	4	4	2	4	4	2
Tigecyline	<0.125	<0.125			<0.125		
Moxifloxacin	>2	>2			>2		
Gentamycin High level	>500	>500			>500		
Mupirocin	256	256			256		
Trimethoprim/Sulfamethoxazole	0.5/9.5	0.5/9.5			0.5/9.5		
Erythromycin/Clindamycin	>4/0.5	>4/0.5			>4/0.5		
Clindamycin	>0.5	>0.5			>0.5		
Fusidic acid	>2	>2			>2		
Gentamycin	>8	>8			>8		
Erythromycin	>4	>4			>4		

DSMZ 17050

	Initial	1 *g*			sim. µ*g*		
Antibiotic	Rep. 1	Rep. 1	Rep. 2	Rep. 3	Rep. 1	Rep. 2	Rep. 3
Penicillin G	>8	>8			>8		
Ampicillin	>16	>16			>16		
Ceftarolin	>2	>2			>2		
Oxacillin	>16	>16			>16		
Rifampicin	0.0625	0.0625			0.5	0.0625	
Cefoxitin	>16	>16			>16		
Teicoplanin	>16	>16			>16		
Vancomycin	>32	>32			>32		
Fosfomycin	<8	64			64		
Synercid	1	2			2		
Linezolid	<1	<1			<1		
Daptomycin	2	<2			2	1	
Tigecyline	<0.125	<0.125			<0.125		
Moxifloxacin	1	0.25			1	<0.25	
Gentamycin High level	<128	<128			500	<128	
Mupirocin	<1	256			256		
Trimethoprim/Sulfamethoxazole	0.5/9.5	0.03125/0.59375			0.03125/0.59375		
Erythromycin/Clindamycin	>4/0.5	>4/0.5			>4/0.5		
Clindamycin	>0.5	>0.5			>0.5		
Fusidic acid	<1	<1			>2		
Gentamycin	4	8			4		
Erythromycin	>4	>4			>4		

VVE-B-16

<table>
<tr><th></th><th>Initial</th><th colspan="3">1 *g*</th><th colspan="3">sim. µ*g*</th></tr>
<tr><th>Antibiotic</th><th>Rep. 1</th><th>Rep. 1</th><th>Rep. 2</th><th>Rep. 3</th><th>Rep. 1</th><th>Rep. 2</th><th>Rep. 3</th></tr>
<tr><td>Penicillin G</td><td>>8</td><td colspan="3">>8</td><td colspan="3">>8</td></tr>
<tr><td>Ampicillin</td><td>>16</td><td colspan="3">>16</td><td colspan="3">>16</td></tr>
<tr><td>Ceftarolin</td><td>>2</td><td colspan="3">>2</td><td colspan="3">>2</td></tr>
<tr><td>Oxacillin</td><td>>16</td><td colspan="3">>16</td><td colspan="3">>16</td></tr>
<tr><td>Rifampicin</td><td>>2</td><td colspan="3">>2</td><td colspan="3">>2</td></tr>
<tr><td>Cefoxitin</td><td>>16</td><td colspan="3">>16</td><td colspan="3">>16</td></tr>
<tr><td>Teicoplanin</td><td>0.5</td><td colspan="3">0.5</td><td colspan="3">0.5</td></tr>
<tr><td>Vancomycin</td><td>0.5</td><td colspan="3">/</td><td colspan="3">0.5</td></tr>
<tr><td>Fosfomycin</td><td>64</td><td colspan="3">64</td><td colspan="3">>64</td></tr>
<tr><td>Synercid</td><td>1</td><td>1</td><td>2</td><td>2</td><td>1</td><td>2</td><td>2</td></tr>
<tr><td>Linezolid</td><td><1</td><td><1</td><td><1</td><td>2</td><td colspan="3">2</td></tr>
<tr><td>Daptomycin</td><td>2</td><td colspan="3">4</td><td colspan="3">4</td></tr>
<tr><td>Tigecyline</td><td><0.125</td><td colspan="3"><0.125</td><td colspan="3"><0.125</td></tr>
<tr><td>Moxifloxacin</td><td>>2</td><td colspan="3">>2</td><td colspan="3">>2</td></tr>
<tr><td>Gentamycin High level</td><td><128</td><td colspan="3"><128</td><td colspan="3"><128</td></tr>
<tr><td>Mupirocin</td><td>256</td><td colspan="3">256</td><td colspan="3">256</td></tr>
<tr><td>Trimethoprim/Sulfamethoxazole</td><td>0.03125/0.59375</td><td colspan="3">0.03125/0.59375</td><td colspan="3">0.03125/0.59375</td></tr>
<tr><td>Erythromycin/Clindamycin</td><td>>4/0.5</td><td colspan="3">>4/0.5</td><td colspan="3">>4/0.5</td></tr>
<tr><td>Clindamycin</td><td><0.5</td><td colspan="3"><0.5</td><td colspan="3"><0.5</td></tr>
<tr><td>Fusidic acid</td><td>>2</td><td colspan="3">2</td><td>>2</td><td>2</td><td>2</td></tr>
<tr><td>Gentamycin</td><td>1</td><td colspan="3">2</td><td colspan="3">2</td></tr>
<tr><td>Erythromycin</td><td><0.25</td><td colspan="3">>4</td><td colspan="3">>4</td></tr>
</table>

VVE-B-17

	**Initial**	**1 *g***			**sim. µ*g***		
Antibiotic	Rep. 1	Rep. 1	Rep. 2	Rep. 3	Rep. 1	Rep. 2	Rep. 3
Penicillin G	>8	>8			>8		
Ampicillin	>16	>16			>16		
Ceftarolin	>2	>2			>2		
Oxacillin	>16	>16			>16		
Rifampicin	>2	>2			>2		
Cefoxitin	>16	>16			>16		
Teicoplanin	0.5	0.5			0.5		
Vancomycin	8	1			32		
Fosfomycin	64	64			64		
Synercid	<0.5	<0.5			1		
Linezolid	<1	<1			<1		
Daptomycin	2	4			4		
Tigecyline	<0.125	<0.125			<0.125		
Moxifloxacin	>2	>2			2		
Gentamycin High level	<128	<128			<128		
Mupirocin	>1	256			256		
Trimethoprim/Sulfamethoxazole	0.03125/0.59375	0.5/9.5			0.5/9.5		
Erythromycin/Clindamycin	4/0.5	4/0.5			4/0.5		
Clindamycin	0.5	0.5			0.5		
Fusidic acid	>2	2			>2		
Gentamycin	2	4			2		
Erythromycin	>4	4			4		

VVE-B-18

	**Initial**	**1 *g***			**sim. µ*g***		
Antibiotic	Rep. 1	Rep. 1	Rep. 2	Rep. 3	Rep. 1	Rep. 2	Rep. 3
Penicillin G	>8	>8			>8		
Ampicillin	>16	>16			>16		
Ceftarolin	>2	>2			>2		
Oxacillin	>16	>16			>16		
Rifampicin	0.5	>2			>2		
Cefoxitin	>16	>16			>16		
Teicoplanin	0.5	1			0.5		
Vancomycin	32	32			32		
Fosfomycin	64	64			64		
Synercid	<0.5	<0.5			<0.5		
Linezolid	2	<1			<1		
Daptomycin	2	4			4		
Tigecyline	<0.125	<0.125			<0.125		
Moxifloxacin	>2	>2			2		
Gentamycin High level	<128	<128			<128		
Mupirocin	256	256			256		
Trimethoprim/Sulfamethoxazole	0.03125/0.59375	0.03125/0.59375			0.03125/0.59375		
Erythromycin/Clindamycin	4/0.5	4/0.5			4/0.5		
Clindamycin	0.5	0.5			0.5		
Fusidic acid	2	2			>2		
Gentamycin	>8	>8			>8		
Erythromycin	>4	>4			>4		

VVE-B-19

	Initial	1 *g*			sim. µ*g*		
Antibiotic	Rep. 1	Rep. 1	Rep. 2	Rep. 3	Rep. 1	Rep. 2	Rep. 3
Penicillin G	>8	>8			>8		
Ampicillin	>16	>16			>16		
Ceftarolin	>2	>2			>2		
Oxacillin	>16	>16			>16		
Rifampicin	2	>2			>2		
Cefoxitin	>16	>16			>16		
Teicoplanin	0.5	0.5			0.5		
Vancomycin	2	32			32		
Fosfomycin	64	>64	64	64	64	>64	>64
Synercid	<0.5	<0.5			<0.5		
Linezolid	<1	<1			<1		
Daptomycin	1	4	2	2	2		
Tigecyline	<0.125	<0.125			<0.125		
Moxifloxacin	>2	>2			>2		
Gentamycin High level	<128	<128			<128		
Mupirocin	<1	256			256		
Trimethoprim/Sulfamethoxazole	0.03125/0.59375	0.03125/0.59375			0.03125/0.59375		
Erythromycin/Clindamycin	>4/0.5	>4/0.5			>4/0.5		
Clindamycin	>0.5	>0.5			>0.5		
Fusidic acid	>2	>2			>2		
Gentamycin	2	2			1	2	2
Erythromycin	>4	>4			>4		

VVE-B-20

	Initial	1 *g*			sim. µ*g*		
Antibiotic	Rep. 1	Rep. 1	Rep. 2	Rep. 3	Rep. 1	Rep. 2	Rep. 3
Penicillin G	>8	>8			>8		
Ampicillin	>16	>16			>16		
Ceftarolin	>2	>2			>2		
Oxacillin	>16	>16			>16		
Rifampicin	0.5	>2			>2		
Cefoxitin	>16	>16			>16		
Teicoplanin	0.25	0.5			0.5		
Vancomycin	8	32			32		
Fosfomycin	>64	>64	64		>64		
Synercid	<0.5	<0.5	1		<0.5		
Linezolid	<1	<1			<1		
Daptomycin	<0.5	2			2		
Tigecyline	<0.125	<0.125			<0.125		
Moxifloxacin	>2	>2			>2		
Gentamycin High level	<128	<128			<128		
Mupirocin	<1	256			256		
Trimethoprim/Sulfamethoxazole	0.03125/0.59375	0.03125/0.59375			0.03125/0.59375		
Erythromycin/Clindamycin	>4/0.5	>4/0.5			>4/0.5		
Clindamycin	>0.5	>0.5			>0.5		
Fusidic acid	>2	>2			>2		
Gentamycin	1	2			2		
Erythromycin	>4	>4			>4		

VVE-B-21

	Initial	1 *g*			sim. µ*g*		
Antibiotic	Rep. 1	Rep. 1	Rep. 2	Rep. 3	Rep. 1	Rep. 2	Rep. 3
Penicillin G	>8	>8			>8		
Ampicillin	>16	>16			>16		
Ceftarolin	>2	>2			>2		
Oxacillin	>16	>16			>16		
Rifampicin	>2	2	0.5	0.5	>2		
Cefoxitin	>16	>16			>16		
Teicoplanin	0.5	1			0.5		
Vancomycin	>32	>32			>32		
Fosfomycin	>64	>64			>64		
Synercid	<0.5	>4			<0.5		
Linezolid	<1	<1			<1		
Daptomycin	4	4			4		
Tigecyline	<0.125	<0.125			<0.125		
Moxifloxacin	>2	>2			>2		
Gentamycin High level	<128	<128			<128		
Mupirocin	256	256			256		
Trimethoprim/Sulfamethoxazole	0.03125/0.59375	0.03125/0.59375			0.03125/0.59375		
Erythromycin/Clindamycin	>4/0.5	>4/0.5			>4/0.5		
Clindamycin	>0.5	>0.5			>0.5		
Fusidic acid	>2	>2			>2		
Gentamycin	>8	>8			>8	8	>8
Erythromycin	>4	>4			>4		

VVE-B-22

	Initial	1 *g*			sim. µ*g*		
Antibiotic	Rep. 1	Rep. 1	Rep. 2	Rep. 3	Rep. 1	Rep. 2	Rep. 3
Penicillin G	>8	>8			>8		
Ampicillin	>16	>16			>16		
Ceftarolin	>2	>2			>2		
Oxacillin	>16	>16			>16		
Rifampicin	0.5	0.5			>2		
Cefoxitin	>16	>16			>16		
Teicoplanin	0.5	1	2	2	1	0.5	0.5
Vancomycin	>32	>32			>32		
Fosfomycin	>64	>64	64	>64	64	>64	>64
Synercid	<0.5	>4	4	<0.5	<0.5		
Linezolid	<1	<1			<1		
Daptomycin	2	4			4		
Tigecyline	<0.125	<0.125			<0.125		
Moxifloxacin	>2	>2			>2		
Gentamycin High level	<128	<128			<128		
Mupirocin	256	256			256		
Trimethoprim/Sulfamethoxazole	0.03125/0.59375	0.03125/0.59375			0.03125/0.59375		
Erythromycin/Clindamycin	>4/0.5	>4/0.5			>4/0.5		
Clindamycin	>0.5	>0.5			>0.5		
Fusidic acid	>2	>2			<1	>2	>2
Gentamycin	8	8			<0.5	8	8
Erythromycin	>4	>4			<0.25	>4	>4

VSE-23

	Initial	1 *g*			sim. µ*g*		
Antibiotic	Rep. 1	Rep. 1	Rep. 2	Rep. 3	Rep. 1	Rep. 2	Rep. 3
Penicillin G	>8	>8			>8		
Ampicillin	>16	>16			>16		
Ceftarolin	>2	>2			>2		
Oxacillin	>16	>16			>16		
Rifampicin	>2	>2			>2		
Cefoxitin	>16	>16			>16		
Teicoplanin	0.5	0.5			0.5		
Vancomycin	0.5	0.5			0.5	0.5	0.5
Fosfomycin	64	64			64		
Synercid	<0. 5	<0. 5			<0.5	<0.5	1
Linezolid	<1	2	<1	<1	<1	2	2
Daptomycin	4	4			4		
Tigecyline	<0.125	<0.125			<0.125		
Moxifloxacin	>2	>2			2		
Gentamycin High level	128	128			128		
Mupirocin	>1	256			256		
Trimethoprim/Sulfamethoxazole	0.03125/0.59375	0.5/9.5			<0.125		
Erythromycin/Clindamycin	4/0.5	4/0.5			4/0.5		
Clindamycin	0.5	0.5			0.5		
Fusidic acid	>2	2			>2		
Gentamycin	>8	8			8		
Erythromycin	>4	4			4		

VSE-24

	Initial	1 *g*			sim. µ*g*		
Antibiotic	Rep. 1	Rep. 1	Rep. 2	Rep. 3	Rep. 1	Rep. 2	Rep. 3
Penicillin G	>8	>8			>8		
Ampicillin	>16	>16			>16		
Ceftarolin	>2	>2			>2		
Oxacillin	>16	>16			>16		
Rifampicin	>2	>2			>2		
Cefoxitin	>16	>16			>16		
Teicoplanin	0.5	0.5			0.5		
Vancomycin	0.5	2	1	1	0.5		
Fosfomycin	>64	64			64		
Synercid	2	4			4		
Linezolid	2	<1	<1	2	2		
Daptomycin	2	4	2	1	4		
Tigecyline	<0.125	<0.125			<0.125		
Moxifloxacin	>2	>2			2		
Gentamycin High level	<128	<128			<128		
Mupirocin	256	256			256		
Trimethoprim/Sulfamethoxazole	0.03125/0.59375	0.03125/0.59375			0.03125/0.59375		
Erythromycin/Clindamycin	4/0.5	4/0.5			4/0.5		
Clindamycin	0.5	0.5			0.5		
Fusidic acid	2	2			>2		
Gentamycin	8	8	4	8	1		
Erythromycin	>4	4			4		

VSE-25

	Initial	1 *g*			sim. µ*g*		
Antibiotic	Rep. 1	Rep. 1	Rep. 2	Rep. 3	Rep. 1	Rep. 2	Rep. 3
Penicillin G	>8	4	2	2	4	1	1
Ampicillin	>16	<2			<2		
Ceftarolin	>2	0.5			1	<0.25	0.5
Oxacillin	>16	>16			>16	8	>16
Rifampicin	1	>2			>2		
Cefoxitin	>16	>16			>16		
Teicoplanin	0.5	0.5			0.5	0.25	0.25
Vancomycin	2	2			2	1	2
Fosfomycin	64	64	>64	>64	32	64	64
Synercid	1	<0.5			1		
Linezolid	<1	<1			<1		
Daptomycin	4	4	>4	>4	4		
Tigecyline	<0.125	<0.125			<0.125		
Moxifloxacin	>2	>2			>2		
Gentamycin High level	<128	<128			<128		
Mupirocin	256	256			256		
Trimethoprim/Sulfamethoxazole	0.03125/0.59375	0.03125/0.59375			0.03125/0.59375		
Erythromycin/Clindamycin	>4/0.5	>4/0.5			>4/0.5		
Clindamycin	>0.5	>0.5			>0.5		
Fusidic acid	>2	>2	2	2	2	2	>2
Gentamycin	4	2			2		
Erythromycin	>4	2	4		>4	2	4

VSE-26

Antibiotic	Initial	1 *g*			sim. µ*g*		
	Rep. 1	Rep. 1	Rep. 2	Rep. 3	Rep. 1	Rep. 2	Rep. 3
Penicillin G	>8	2			2		
Ampicillin	>16	<2			<2		
Ceftarolin	>2	2			2		
Oxacillin	>16	16			8	16	
Rifampicin	2	1			0.5	2	
Cefoxitin	>16	>16			>16		
Teicoplanin	0.25	0.5			0.25	0.5	
Vancomycin	1	1			1		
Fosfomycin	>64	>64	64		>64		
Synercid	<0.5	>4			>4		
Linezolid	<1	<1			<1		
Daptomycin	2	4	2		>4	2	
Tigecyline	<0.125	<0.125			<0.125		
Moxifloxacin	>2	>2			>2		
Gentamycin High level	<128	<128			<128		
Mupirocin	256	256			256		
Trimethoprim/Sulfamethoxazole	0.03125/0.59375	0.03125/0.59375			0.03125/0.59375		
Erythromycin/Clindamycin	>4/0.5	>4/0.5			4/0.5		
Clindamycin	>0.5	>0.5			>0.5		
Fusidic acid	<1	<1			<1	<1	
Gentamycin	4	2			1	2	
Erythromycin	>4	>4			>4		

VSE-27

<table>
<tr><th></th><th>Initial</th><th colspan="3">1 g</th><th colspan="3">sim. µg</th></tr>
<tr><th>Antibiotic</th><th>Rep. 1</th><th>Rep. 1</th><th>Rep. 2</th><th>Rep. 3</th><th>Rep. 1</th><th>Rep. 2</th><th>Rep. 3</th></tr>
<tr><td>Penicillin G</td><td>>8</td><td colspan="3">>8</td><td colspan="3">>8</td></tr>
<tr><td>Ampicillin</td><td>>16</td><td colspan="3">>16</td><td colspan="3">>16</td></tr>
<tr><td>Ceftarolin</td><td>>2</td><td colspan="3">>2</td><td colspan="3">>2</td></tr>
<tr><td>Oxacillin</td><td>>16</td><td colspan="3">>16</td><td colspan="3">>16</td></tr>
<tr><td>Rifampicin</td><td>>2</td><td colspan="3">>2</td><td colspan="3">>2</td></tr>
<tr><td>Cefoxitin</td><td>>16</td><td colspan="3">>16</td><td colspan="3">>16</td></tr>
<tr><td>Teicoplanin</td><td>0.5</td><td colspan="3">0.5</td><td>1</td><td colspan="2">0.5</td></tr>
<tr><td>Vancomycin</td><td>1</td><td colspan="3">1</td><td colspan="3">1</td></tr>
<tr><td>Fosfomycin</td><td>>64</td><td>>64</td><td>64</td><td>>64</td><td></td><td colspan="2">>64</td></tr>
<tr><td>Synercid</td><td><0.5</td><td colspan="3"><0.5</td><td colspan="3"><0.5</td></tr>
<tr><td>Linezolid</td><td>1</td><td colspan="3"><1</td><td colspan="3"><1</td></tr>
<tr><td>Daptomycin</td><td>4</td><td colspan="3">4</td><td colspan="3">4</td></tr>
<tr><td>Tigecyline</td><td><0.125</td><td colspan="3"><0.125</td><td colspan="3"><0.125</td></tr>
<tr><td>Moxifloxacin</td><td>>2</td><td colspan="3">>2</td><td colspan="3">>2</td></tr>
<tr><td>Gentamycin High level</td><td>>500</td><td colspan="3">>500</td><td colspan="3">>500</td></tr>
<tr><td>Mupirocin</td><td>1</td><td colspan="3">256</td><td colspan="3">256</td></tr>
<tr><td>Trimethoprim/Sulfamethoxazole</td><td>0.03125/0.59375</td><td colspan="3">>4/76</td><td colspan="3">>4/76</td></tr>
<tr><td>Erythromycin/Clindamycin</td><td>>4/0.5</td><td colspan="3">>4/0.5</td><td colspan="3">>4/0.5</td></tr>
<tr><td>Clindamycin</td><td>>0.5</td><td colspan="3">>0.5</td><td colspan="3">>0.5</td></tr>
<tr><td>Fusidic acid</td><td>>2</td><td colspan="3">>2</td><td colspan="3">>2</td></tr>
<tr><td>Gentamycin</td><td>>8</td><td colspan="3">>8</td><td colspan="3">>8</td></tr>
<tr><td>Erythromycin</td><td>>4</td><td colspan="3">>4</td><td colspan="3">>4</td></tr>
</table>

VSE-28

	Initial	1 *g*			sim. µ*g*		
Antibiotic	Rep. 1	Rep. 1	Rep. 2	Rep. 3	Rep. 1	Rep. 2	Rep. 3
Penicillin G	>8	>8			>8		
Ampicillin	>16	>16			>16		
Ceftarolin	>2	>2			>2		
Oxacillin	>16	>16			>16		
Rifampicin	>2	>2			>2		
Cefoxitin	>16	>16			>16		
Teicoplanin	0.5	0.5			0.5	0.5	0.25
Vancomycin	0.5	1	0.5	1	0.5	1	0.5
Fosfomycin	64	>64	64	64	64		
Synercid	<0.5	<0.5			<0.5		
Linezolid	<1	<1			<1		
Daptomycin	2	2			2	2	4
Tigecyline	<0.125	<0.125			<0.125		
Moxifloxacin	>2	>2			>2		
Gentamycin High level	<128	<128			<128		
Mupirocin	1	256			256		
Trimethoprim/Sulfamethoxazole	<0.03125/0.59375	<0.03125/0.59375			<0.03125/0.59375		
Erythromycin/Clindamycin	>4/0.5	>4/0.5			>4/0.5		
Clindamycin	>0.5	>0.5			>0.5		
Fusidic acid	>2	>2			>2		
Gentamycin	1	1			1		
Erythromycin	>4	>4			>4		

VSE-29

<table>
<tr><th></th><th>Initial</th><th colspan="3">1 g</th><th colspan="3">sim. µg</th></tr>
<tr><th>Antibiotic</th><th></th><th>Rep. 1</th><th>Rep. 2</th><th>Rep. 3</th><th>Rep. 1</th><th>Rep. 2</th><th>Rep. 3</th></tr>
<tr><td>Penicillin G</td><td>>8</td><td colspan="3">>8</td><td colspan="3">>8</td></tr>
<tr><td>Ampicillin</td><td>>16</td><td colspan="3">>16</td><td colspan="3">>16</td></tr>
<tr><td>Ceftarolin</td><td>>2</td><td colspan="3">>2</td><td colspan="3">>2</td></tr>
<tr><td>Oxacillin</td><td>>16</td><td colspan="3">>16</td><td colspan="3">>16</td></tr>
<tr><td>Rifampicin</td><td>>2</td><td colspan="3">>2</td><td colspan="3">>2</td></tr>
<tr><td>Cefoxitin</td><td>>16</td><td colspan="3">>16</td><td colspan="3">>16</td></tr>
<tr><td>Teicoplanin</td><td>0.5</td><td colspan="3">0.5</td><td colspan="3">0.5</td></tr>
<tr><td>Vancomycin</td><td>0.5</td><td colspan="3">0.5</td><td>0.5</td><td>1</td><td>1</td></tr>
<tr><td>Fosfomycin</td><td>>64</td><td colspan="3">>64</td><td colspan="3">>64</td></tr>
<tr><td>Synercid</td><td><0.5</td><td colspan="3"><0.5</td><td>2</td><td>0.5</td><td>0.5</td></tr>
<tr><td>Linezolid</td><td><1</td><td colspan="3"><1</td><td colspan="3"><1</td></tr>
<tr><td>Daptomycin</td><td>2</td><td colspan="3">2</td><td colspan="3">2</td></tr>
<tr><td>Tigecyline</td><td><0.125</td><td colspan="3"><0.125</td><td colspan="3"><0.125</td></tr>
<tr><td>Moxifloxacin</td><td>>2</td><td colspan="3">>2</td><td colspan="3">>2</td></tr>
<tr><td>Gentamycin High level</td><td><128</td><td colspan="3"><128</td><td colspan="3"><128</td></tr>
<tr><td>Mupirocin</td><td><1</td><td colspan="3">256</td><td colspan="3">256</td></tr>
<tr><td>Trimethoprim/Sulfamethoxazole</td><td>0.03125/0.59375</td><td colspan="3">0.03125/0.59375</td><td colspan="3">0.03125/0.59375</td></tr>
<tr><td>Erythromycin/Clindamycin</td><td>>4/0.5</td><td colspan="3">>4/0.5</td><td colspan="3">>4/0.5</td></tr>
<tr><td>Clindamycin</td><td>>0.5</td><td colspan="3">>0.5</td><td colspan="3">>0.5</td></tr>
<tr><td>Fusidic acid</td><td>>2</td><td colspan="3">>2</td><td colspan="3">>2</td></tr>
<tr><td>Gentamycin</td><td>2</td><td colspan="3">2</td><td>1</td><td>2</td><td>1</td></tr>
<tr><td>Erythromycin</td><td>>4</td><td colspan="3">>4</td><td colspan="3">>4</td></tr>
</table>

VSE-30

	Initial	1 *g*			sim. µ*g*		
Antibiotic	Rep 1	Rep. 1	Rep. 2	Rep. 3	Rep. 1	Rep. 2	Rep. 3
Penicillin G	>8	>8			>8		
Ampicillin	>16	>8			>16		
Ceftarolin	>2	>2			>2		
Oxacillin	>16	>16			>16		
Rifampicin	>2	>2			>2		
Cefoxitin	>16	>16			>16		
Teicoplanin	0.5	0.5			0.5		
Vancomycin	0.5	0.5			1		
Fosfomycin	64	<8	>64	>65	>64		
Synercid	<0.5	<0.5			<0.5		
Linezolid	2	2			<1		
Daptomycin	2	4	2	2	4	2	2
Tigecyline	<0.125	<0.125			<0.125		
Moxifloxacin	>2	<0.25	>2	>2	>2		
Gentamycin High level	>500	<128			>500	<128	<128
Mupirocin	256	256			256		
Trimethoprim/Sulfamethoxazole	0.03125/0.59375	0.5/9.5	0.03125/0.59375		0.03125/0.59375		
Erythromycin/Clindamycin	>4/0.5	>4/0.5			>4/0.5		
Clindamycin	>0.5	>0.5			>0.5		
Fusidic acid	>2	>2			>2		
Gentamycin	2	4			2		
Erythromycin	>4	>4			>4		

VSE-31

	Initial	1 *g*			sim. µ*g*		
Antibiotic	Rep. 1	Rep. 1	Rep. 2	Rep. 3	Rep. 1	Rep. 2	Rep. 3
Penicillin G	>8	>8			>8		
Ampicillin	>16	>16			>16		
Ceftarolin	>2	>2			>2		
Oxacillin	>16	>16			>16		
Rifampicin	0.5	0.5	1	0.5	0.5	0.5	>2
Cefoxitin	>16	>16			>16		
Teicoplanin	0.25	0.25			0.25		
Vancomycin	0.5	0.5	0.5	1	0.5	0.5	1
Fosfomycin	64	<8	64	64	>64	>64	64
Synercid	2	4			4		
Linezolid	<1	<1			<1		
Daptomycin	2	2			2		
Tigecyline	<0.125	<0.125			<0.125		
Moxifloxacin	>2	>2			>2		
Gentamycin High level	>500	>500	>500	500	>500		
Mupirocin	256	256	256	<1	256		
Trimethoprim/Sulfamethoxazole	0.03125/0.59375	0.03125/0.59375			0.03125/0.59375		
Erythromycin/Clindamycin	>4/0.5	>4/0.5			>4/0.5		
Clindamycin	>0.5	>0.5			>0.5		
Fusidic acid	>2	>2	>2	<1	>2		
Gentamycin	>8	>8	>8	4	>8		
Erythromycin	>4	>4	>4	1	>4		

VSE-32

	Initial	1 *g*			sim. µ*g*		
Antibiotic	Rep. 1	Rep. 1	Rep. 2	Rep. 3	Rep. 1	Rep. 2	Rep. 3
Penicillin G	>8	>8			>8		
Ampicillin	>16	>16			>16		
Ceftarolin	>2	>2			>2		
Oxacillin	>16	>16			>16		
Rifampicin	0.5	>2	0.5	>2	>2		
Cefoxitin	>16	>16			>16		
Teicoplanin	0.5	1	0.5	1	0.5		
Vancomycin	0.5	1			0.5		
Fosfomycin	64	64	64	>64	64	64	>64
Synercid	<0.5	<0.5			<0.5		
Linezolid	<1	<1			<1		
Daptomycin	2	2			2		
Tigecyline	<0.125	<0.125			<0.125		
Moxifloxacin	>2	>2			>2		
Gentamycin High level	<128	<128	>500	<128	>500	<128	>500
Mupirocin	>256	256			<1	256	256
Trimethoprim/Sulfamethoxazole	0.5/9.5	0.5/9.5			0.5/9.5		
Erythromycin/Clindamycin	>4/0.5	>4/0.5			>4/0.5		
Clindamycin	>0.5	>0.5			>0.5		
Fusidic acid	>2	>2			>2		
Gentamycin	1	1	2	2	1	1	2
Erythromycin	>4	>4			>4		

VSE-33

	Initial	1 *g*			sim. µ*g*		
Antibiotic	Rep. 1	Rep. 1	Rep. 2	Rep. 3	Rep. 1	Rep. 2	Rep. 3
Penicillin G	>8	>8			>8		
Ampicillin	>16	>16			>16		
Ceftarolin	>2	>2			>2		
Oxacillin	>16	>16			>16		
Rifampicin	>2	>2			>2		
Cefoxitin	>16	>16			>16		
Teicoplanin	0.5	0.5			0.5		
Vancomycin	1	1			1		
Fosfomycin	>64	>64			>64		
Synercid	1	1			1		
Linezolid	<1	2			2		
Daptomycin	4	>4			>4		
Tigecyline	<0.125	<0.125			<0.125		
Moxifloxacin	>2	>2			>2		
Gentamycin High level	<128	500	<128		<128		
Mupirocin	256	256			256		
Trimethoprim/Sulfamethoxazole	0.03125/0.59375	0.03125/ 0.59375	0.5/9.5		0.03125/0.59375		
Erythromycin/Clindamycin	>4/0.5	>4/0.5			>4/0.5		
Clindamycin	>0.5	>0.5			<0.5		
Fusidic acid	>2	>2			>2		
Gentamycin	2	2			2		
Erythromycin	>4	>4			>4		

VSE-34

Antibiotic	Initial	1 *g*			sim. µ*g*		
	Rep. 1	Rep. 1	Rep. 2	Rep. 3	Rep. 1	Rep. 2	Rep. 3
Penicillin G	>8	>8			>8		
Ampicillin	>16	>16			>16		
Ceftarolin	>2	>2			>2		
Oxacillin	>16	>16			>16		
Rifampicin	>2	>2			>2		
Cefoxitin	>16	>16			>16		
Teicoplanin	0.5	0.5			0.5		
Vancomycin	0.5	1			0.5	1	1
Fosfomycin	64	>64			>64		
Synercid	1	2			1		
Linezolid	2	4	2		2		
Daptomycin	4	4			4		
Tigecyline	<0.125	<0.125			<0.125		
Moxifloxacin	>2	>2			>2		
Gentamycin High level	>500	>500			>500		
Mupirocin	256	256			256		
Trimethoprim/Sulfamethoxazole	0.03125/0.59375	0.03125/0.59375			0.03125/0.59375		
Erythromycin/Clindamycin	<4/0.5	4/0.5			4/0.5		
Clindamycin	<0.5	<0.5			<0.5		
Fusidic acid	>2	>2			>2		
Gentamycin	>8	>8			4		
Erythromycin	>4	>4			>4		

VSE-35

	Initial	1 *g*			sim. µ*g*		
Antibiotic	Rep. 1	Rep. 1	Rep. 2	Rep. 3	Rep. 1	Rep. 2	Rep. 3
Penicillin G	>8	2			2		
Ampicillin	>16	<2			<2		
Ceftarolin	>2	0.5			1		
Oxacillin	>16	8	>16	>16	8		
Rifampicin	1	0.0625			0.0625		
Cefoxitin	>16	>16			>16		
Teicoplanin	0.25	0.25	0.5	0.25	0.25		
Vancomycin	8	0.5			0.5		
Fosfomycin	32	>64	64	64	>64		
Synercid	1	2			1		
Linezolid	<1	<1			<1		
Daptomycin	4	2	2	4	2		
Tigecyline	<0.125	<0.125			<0.125		
Moxifloxacin	>2	0.5			<0.25		
Gentamycin High level	<128	<128			<128		
Mupirocin	256	256			256		
Trimethoprim/Sulfamethoxazole	0.03125/0.59375	<0.03125/0.59375			<0.03125/0.59375		
Erythromycin/Clindamycin	>4/0.5	<4/0.5			<4/0.5		
Clindamycin	>0.5	>0.5			>0.5		
Fusidic acid	<1	>2			>2		
Gentamycin	2	2			2		
Erythromycin	>4	>4			>4		

ATCC 6057

<table>
<tr><th></th><th>Initial</th><th colspan="3">1 *g*</th><th colspan="3">sim. µ*g*</th></tr>
<tr><th>Antibiotic</th><th>Rep. 1</th><th>Rep. 1</th><th>Rep. 2</th><th>Rep. 3</th><th>Rep. 1</th><th>Rep. 2</th><th>Rep. 3</th></tr>
<tr><td>Penicillin G</td><td>>8</td><td colspan="3">>8</td><td colspan="3">>8</td></tr>
<tr><td>Ampicillin</td><td>8</td><td>>16</td><td colspan="2">8</td><td colspan="3">>16</td></tr>
<tr><td>Ceftarolin</td><td>>2</td><td colspan="3">>2</td><td colspan="3">>2</td></tr>
<tr><td>Oxacillin</td><td>>16</td><td colspan="3">>16</td><td colspan="3">>2</td></tr>
<tr><td>Rifampicin</td><td>0.0625</td><td colspan="3">0.0625</td><td colspan="3">0.0625</td></tr>
<tr><td>Cefoxitin</td><td>>16</td><td colspan="3">>16</td><td colspan="3">>16</td></tr>
<tr><td>Teicoplanin</td><td>>16</td><td colspan="3">>16</td><td colspan="3">>16</td></tr>
<tr><td>Vancomycin</td><td>>32</td><td colspan="3">>32</td><td colspan="3">>32</td></tr>
<tr><td>Fosfomycin</td><td>32</td><td colspan="3">32</td><td colspan="3">64</td></tr>
<tr><td>Synercid</td><td>1</td><td colspan="3"><0.5</td><td><0.5</td><td>1</td><td>1</td></tr>
<tr><td>Linezolid</td><td><1</td><td colspan="3"><1</td><td colspan="3"><1</td></tr>
<tr><td>Daptomycin</td><td>2</td><td><0.5</td><td colspan="2">2</td><td>2</td><td>1</td><td>2</td></tr>
<tr><td>Tigecyline</td><td><0.125</td><td colspan="3"><0.125</td><td colspan="3"><0.125</td></tr>
<tr><td>Moxifloxacin</td><td><1</td><td><0.25</td><td colspan="2">1</td><td>1</td><td>0.5</td><td>1</td></tr>
<tr><td>Gentamycin High level</td><td><128</td><td colspan="3"><128</td><td colspan="3"><128</td></tr>
<tr><td>Mupirocin</td><td><1</td><td colspan="3">256</td><td colspan="3">256</td></tr>
<tr><td>Trimethoprim/Sulfamethoxazole</td><td>0.03125/0.59375</td><td colspan="3"><0.03125/0.59375</td><td colspan="3"><0.03125/0.59375</td></tr>
<tr><td>Erythromycin/Clindamycin</td><td>>4/0.5</td><td colspan="3">>4/0.5</td><td colspan="3">>4/0.5</td></tr>
<tr><td>Clindamycin</td><td>>0.5</td><td colspan="3">>0.5</td><td colspan="3">>0.5</td></tr>
<tr><td>Fusidic acid</td><td><1</td><td colspan="3"><1</td><td colspan="3">>2</td></tr>
<tr><td>Gentamycin</td><td>2</td><td colspan="3">2</td><td>2</td><td>1</td><td>4</td></tr>
<tr><td>Erythromycin</td><td>>4</td><td colspan="3">>4</td><td colspan="3">>4</td></tr>
</table>

VSE-37

<table>
<tr><th></th><th>Initial</th><th colspan="3">1 g</th><th colspan="3">sim. µg</th></tr>
<tr><th>Antibiotic</th><th>Rep. 1</th><th>Rep. 1</th><th>Rep. 2</th><th>Rep. 3</th><th>Rep. 1</th><th>Rep. 2</th><th>Rep. 3</th></tr>
<tr><td>Penicillin G</td><td>>8</td><td colspan="3">>8</td><td colspan="3">>8</td></tr>
<tr><td>Ampicillin</td><td>>16</td><td colspan="3">>16</td><td colspan="3">>16</td></tr>
<tr><td>Ceftarolin</td><td>>2</td><td colspan="3">>2</td><td colspan="3">>2</td></tr>
<tr><td>Oxacillin</td><td>>16</td><td colspan="3">>16</td><td colspan="3">>16</td></tr>
<tr><td>Rifampicin</td><td>>2</td><td colspan="3">>2</td><td colspan="3">>2</td></tr>
<tr><td>Cefoxitin</td><td>>16</td><td colspan="3">>16</td><td colspan="3">>16</td></tr>
<tr><td>Teicoplanin</td><td>0.5</td><td colspan="3">0.5</td><td colspan="3">0.5</td></tr>
<tr><td>Vancomycin</td><td>0.5</td><td colspan="3">0.5</td><td colspan="3">0.5</td></tr>
<tr><td>Fosfomycin</td><td>64</td><td colspan="2">>64</td><td>64</td><td>>64</td><td colspan="2">64</td></tr>
<tr><td>Synercid</td><td><0.5</td><td colspan="3"><0.5</td><td colspan="3"><0.5</td></tr>
<tr><td>Linezolid</td><td><1</td><td colspan="3"><1</td><td colspan="3"><1</td></tr>
<tr><td>Daptomycin</td><td>4</td><td colspan="3">4</td><td colspan="3">4</td></tr>
<tr><td>Tigecyline</td><td><0.125</td><td colspan="3"><0.125</td><td colspan="3"><0.125</td></tr>
<tr><td>Moxifloxacin</td><td>>2</td><td colspan="3">>2</td><td colspan="3">>2</td></tr>
<tr><td>Gentamycin High level</td><td><128</td><td colspan="3"><128</td><td colspan="3"><128</td></tr>
<tr><td>Mupirocin</td><td>256</td><td colspan="3">256</td><td colspan="3">256</td></tr>
<tr><td>Trimethoprim/Sulfamethoxazole</td><td>0.03125/0.59375</td><td colspan="3"><0.03125/0.59375</td><td colspan="3">0.5/9.5</td></tr>
<tr><td>Erythromycin/Clindamycin</td><td><4/0.5</td><td colspan="3"><4/0.5</td><td colspan="3"><4/0.5</td></tr>
<tr><td>Clindamycin</td><td><0.5</td><td colspan="3"><0.5</td><td colspan="3"><0.5</td></tr>
<tr><td>Fusidic acid</td><td>>2</td><td colspan="3">>2</td><td colspan="3">>2</td></tr>
<tr><td>Gentamycin</td><td>2</td><td>2</td><td>1</td><td>8</td><td colspan="3">2</td></tr>
<tr><td>Erythromycin</td><td><0.25</td><td colspan="3"><0.25</td><td colspan="3">0.25</td></tr>
</table>

VSE-38

	Initial	1 *g*			sim. µ*g*		
Antibiotic	Rep. 1	Rep. 1	Rep. 2	Rep. 3	Rep. 1	Rep. 2	Rep. 3
Penicillin G	>8	>8			>8		
Ampicillin	>16	>16			>16		
Ceftarolin	>2	>2			>2		
Oxacillin	>16	>16			>16		
Rifampicin	>2	>2			>2		
Cefoxitin	>16	>16			>16		
Teicoplanin	0.25	0.25			0.25		
Vancomycin	2	1			1		
Fosfomycin	64	>64			>64		
Synercid	<0.5	<0.5	<0.5	4	<0.5		
Linezolid	<1	<1			<1		
Daptomycin	1	1	1	4	2	2	1
Tigecyline	<0.125	<0.125			<0.125		
Moxifloxacin	>2	>2			>2		
Gentamycin High level	<128	>500			<128		
Mupirocin	256	256			256		
Trimethoprim/Sulfamethoxazole	0.03125/0.59375	0.03125/0.59375			0.03125/0.59375		
Erythromycin/Clindamycin	>4/0.5	>4/0.5			>4/0.5		
Clindamycin	>0.5	>0.5			>0.5		
Fusidic acid	>2	>2			>2		
Gentamycin	2	>8			2		
Erythromycin	>4	>4			>4		

VSE-39

Antibiotic	Initial	1 *g*			sim. µ*g*		
	Rep. 1	Rep. 1	Rep. 2	Rep. 3	Rep. 1	Rep. 2	Rep. 3
Penicillin G	>8	>8			>8		
Ampicillin	>16	>16			>16		
Ceftarolin	>2	>2			>2		
Oxacillin	>16	>16			>16		
Rifampicin	>2	>2			>2		
Cefoxitin	>16	>16			>16		
Teicoplanin	0.5	1			1		
Vancomycin	0.5	0.5	1	0.5	0.5		
Fosfomycin	32	>64	>64	32	64		
Synercid	2	4			4		
Linezolid	<1	<1			<1		
Daptomycin	2	4			4		
Tigecyline	<0.125	<0.125			<0.125		
Moxifloxacin	>2	>2			>2		
Gentamycin High level	>500	>500			>500		
Mupirocin	<1	256			256		
Trimethoprim/Sulfamethoxazole	0.03125/0.59375	0.03125/0.59375			0.03125/0.59375		
Erythromycin/Clindamycin	>4/0.5	>4/0.5			>4/0.5		
Clindamycin	>0.5	>0.5			>0.5		
Fusidic acid	>2	>2			>2		
Gentamycin	>8	>8			>8		
Erythromycin	>4	>4			>4		

VSE-40

Antibiotic	Initial	1 *g*			sim. μ*g*		
	Rep. 1	Rep. 1	Rep. 2	Rep. 3	Rep. 1	Rep. 2	Rep. 3
Penicillin G	>8	>8			>8		
Ampicillin	>16	>16			>16		
Ceftarolin	>2	>2			>2		
Oxacillin	>16	>16			>16		
Rifampicin	1	>2			>2		
Cefoxitin	>16	>16			>16		
Teicoplanin	0.25	0.25			0.25		
Vancomycin	0.5	1			0.5		
Fosfomycin	64	64			64		
Synercid	<0.5	<0.5			<0.5		
Linezolid	<1	<1			<1		
Daptomycin	2	2			2		
Tigecyline	<0.125	<0.125			<0.125		
Moxifloxacin	>2	>2			>2		
Gentamycin High level	<128	<128			<128		
Mupirocin	<1	256			256		
Trimethoprim/Sulfamethoxazole	0.03125/0.59375	0.03125/0.59375			0.03125/0.59375		
Erythromycin/Clindamycin	>4/0.5	>4/0.5			>4/0.5		
Clindamycin	>0.5	>0.5			>0.5		
Fusidic acid	>2	>2			>2		
Gentamycin	1	1	1	2	1		
Erythromycin	>4	>4			>4		

VSE-41

<table>
<tr><th></th><th>Initial</th><th colspan="3">1 *g*</th><th colspan="3">sim. µ*g*</th></tr>
<tr><th>Antibiotic</th><th>Rep. 1</th><th>Rep. 1</th><th>Rep. 2</th><th>Rep. 3</th><th>Rep. 1</th><th>Rep. 2</th><th>Rep. 3</th></tr>
<tr><td>Penicillin G</td><td>>8</td><td colspan="3">>8</td><td colspan="3">>8</td></tr>
<tr><td>Ampicillin</td><td>>16</td><td colspan="3">>16</td><td colspan="3">>16</td></tr>
<tr><td>Ceftarolin</td><td>>2</td><td colspan="3">>2</td><td colspan="3">>2</td></tr>
<tr><td>Oxacillin</td><td>>16</td><td colspan="3">>16</td><td colspan="3">>16</td></tr>
<tr><td>Rifampicin</td><td>>2</td><td colspan="3">>2</td><td colspan="3">>2</td></tr>
<tr><td>Cefoxitin</td><td>>16</td><td colspan="3">>16</td><td colspan="3">>16</td></tr>
<tr><td>Teicoplanin</td><td>1</td><td colspan="3">1</td><td colspan="3">1</td></tr>
<tr><td>Vancomycin</td><td>0.5</td><td>>32</td><td>1</td><td>1</td><td>>32</td><td>1</td><td>0.5</td></tr>
<tr><td>Fosfomycin</td><td>64</td><td colspan="3">64</td><td colspan="3">64</td></tr>
<tr><td>Synercid</td><td><0.5</td><td colspan="3">1</td><td colspan="3">1</td></tr>
<tr><td>Linezolid</td><td>2</td><td>2</td><td>>1</td><td>>1</td><td colspan="3">2</td></tr>
<tr><td>Daptomycin</td><td>2</td><td colspan="3">4</td><td colspan="2">4</td><td>>4</td></tr>
<tr><td>Tigecyline</td><td><0.125</td><td colspan="3"><0.125</td><td colspan="3"><0.125</td></tr>
<tr><td>Moxifloxacin</td><td>>2</td><td colspan="3">>2</td><td colspan="3">>2</td></tr>
<tr><td>Gentamycin High level</td><td><128</td><td colspan="3"><128</td><td>500</td><td><128</td><td><128</td></tr>
<tr><td>Mupirocin</td><td>256</td><td colspan="3">256</td><td></td><td>256</td><td></td></tr>
<tr><td>Trimethoprim/Sulfamethoxazole</td><td>0.03125/
0.59375</td><td colspan="2">0.5/9.5
0.5/9.5</td><td>0.03125/
0.59375</td><td colspan="3">0.5/
9.5</td></tr>
<tr><td>Erythromycin/Clindamycin</td><td>>4/0.5</td><td colspan="3">>4/0.5</td><td colspan="3">>4/0.5</td></tr>
<tr><td>Clindamycin</td><td>>0.5</td><td colspan="3">>0.5</td><td colspan="3">>0.5</td></tr>
<tr><td>Fusidic acid</td><td>>2</td><td colspan="3">>2</td><td colspan="3">>2</td></tr>
<tr><td>Gentamycin</td><td>4</td><td colspan="3">2</td><td colspan="3">4</td></tr>
<tr><td>Erythromycin</td><td>>4</td><td colspan="3">>4</td><td colspan="3">>4</td></tr>
</table>

VSE-42

Antibiotic	Initial	1 *g*			sim. µ*g*		
	Rep. 1	Rep. 1	Rep. 2	Rep. 3	Rep. 1	Rep. 2	Rep. 3
Penicillin G	>8	>8			>8		
Ampicillin	>16	>16			>16		
Ceftarolin	>2	>2			>2		
Oxacillin	>16	>16			>16		
Rifampicin	>2	>2			>2		
Cefoxitin	>16	>16			>16		
Teicoplanin	0.5	0.5			0.5		
Vancomycin	0.5	1			1		
Fosfomycin	>64	>64			>64		
Synercid	<0.5	<0.5			<0.5	1	
Linezolid	2	<1			<1		
Daptomycin	4	4			4		
Tigecyline	<0.125	<0.125			<0.125		
Moxifloxacin	>2	>2			>2		
Gentamycin High level	<128	<128			<128		
Mupirocin	1	256			256		
Trimethoprim/Sulfamethoxazole	0.03125/0.59375	0.03125/0.59375			0.5/9.5		
Erythromycin/Clindamycin	>4/0.5	>4/0.5			>4/0.5		
Clindamycin	>0.5	>0.5			>0.5		
Fusidic acid	>2	>2			>2		
Gentamycin	2	1	2	2	2		
Erythromycin	>4	>4			>4		
